# Introducing a novel 28S rRNA marker for improved assessment of coral reef biodiversity

**DOI:** 10.7717/peerj.21662

**Published:** 2026-08-24

**Authors:** Gabrielle Martineau, Robert J. Toonen, Molly A. Timmers, Christopher P. Jury, Jan Vicente

**Affiliations:** 1 University of Hawaiʻi at Mānoa, Hawaiʻi Institute of Marine Biology, Kāneʻohe, HI, United States of America; 2 Institute of Research in Immunology and Cancer, Molecular Genetics of Stem Cells Laboratory, Montreal, QC, Canada; 3 National Geographic Society, Pristine Seas, Washington, DC, United States of America; 4 Department of Natural Sciences, Bernice Pauahi Bishop Museum, Honolulu, HI, United States of America

**Keywords:** Metabarcoding, Universal primers, Porifera, Biodiversity survey, Coral reef monitoring

## Abstract

Biodiversity monitoring based on DNA metabarcoding depends on primer performance. Here, we develop a new metabarcoding primer pair that targets a ∼318 bp fragment of the 28S rRNA gene that is highly conserved across branches of the tree of life. We apply these novel primers to detect coral reef biodiversity and perform a direct comparison with the most commonly used metabarcoding marker for coral reefs, targeting the cyctochrome oxidase subunit I (COI) gene (DOI 10.1186/1742-9994-10-34). We show that our novel 28S primers detect a broader taxonomic scope of species across phyla and classes within complex coral reef communities than the Leray-Geller COI primers. We validate species detection, amplification efficiency, and the quantitative potential of these novel primers using a suite of mock and natural complex reef communities. In the natural complex coral reef community experiments, commonly used COI metabarcoding primers detected only 12 phyla (18 classes), whereas our new 28S primer increased detection to 16 phyla (19 classes). We further validate the performance of these new primers using sponges as the only taxonomic group in this natural community for which an exhaustive species-level metabarcoding reference database exists. Sponges are a notoriously challenging group for coral reef metabarcoding studies, and COI detected only 30.9% of the species identified by eye, whereas our primers increased that to 79.4% of species. Mock community experiments detected most of the sponge species present (94/146) spanning a broad taxonomic scope (15 orders), and showed limited taxon-specific primer biases with only a single species exceeding a two-fold deviation from the expected number of reads based on abundance. Limited primer bias allows for possible quantitative metabarcoding, and we further show a significant relationship between read abundance and percent coverage of the sponge taxa identified by eye (*R* = 0.76). As biodiversity assessments using metabarcoding tools are increasingly being leveraged for environmental monitoring and guide policymaking, these new 28S rRNA primers may improve biodiversity assessments for complex ecological coral reef communities.

## Introduction

As climate change increasingly alters ecosystems across the planet, it is critical to document biodiversity before it is lost (Butchart et al., 2010; McCauley et al., 2015; Pimm et al., 2014). Coral reefs, the most diverse and species-rich marine habitat, are among the most severely threatened habitats on the planet (Carpenter et al., 2008; Hoegh-Guldberg et al., 2007). Traditional methods to assess biodiversity of coral reefs (Beijbom et al., 2012; David et al., 2019; Williams et al., 2019) remain time-consuming, expensive, and often rely on sparse and declining taxonomic expertise (Baird & Hajibabaei, 2012; Bik et al., 2012; Elbrecht & Leese, 2015; Taberlet et al., 2012; Yu et al., 2012). As high-throughput sequencing technology has become widely affordable, molecular approaches have proved to be efficient tools for identifying organisms in complex ecological samples (Baird & Hajibabaei, 2012; Bohmann et al., 2014; Elbrecht & Leese, 2017; Leray & Knowlton, 2015; Steyaert et al., 2022; Yu et al., 2012). Amplification of a shared locus from a diverse environmental sample, (Baird & Hajibabaei, 2012; Hajibabaei et al., 2011; Taberlet et al., 2012; Yu et al., 2012), termed DNA metabarcoding, has revolutionized ecological biodiversity monitoring with direct implications for conservation and management (Ji et al., 2013).

Molecular assessments based on binary (presence/absence) data from biodiversity surveys using metabarcoding have consistently proven to be repeatable and reliable (Aylagas et al., 2016; DiBattista et al., 2019; DiBattista et al., 2020; Elbrecht & Leese, 2015; Nichols & Marko, 2019; Stat et al., 2017). Whether quantitative metrics derived from metabarcoding are sufficiently robust, measures of relative abundance within natural communities remains a subject of considerable debate (Elbrecht & Leese, 2015; Hänfling et al., 2016; Harper et al., 2018; Leray & Knowlton, 2017; Pont et al., 2018; Stoeckle, Soboleva & Charlop-Powers, 2017; Ushio et al., 2017). Primer amplification biases and sequencing errors impact quantitative estimates of community composition based on relative read counts (Deagle et al., 2014; Leray et al., 2013; Piñol, Senar & Symondson, 2019). Because samples include a tiny fraction of the habitat, the factors influencing amplification efficiency are exacerbated by stochasticity in sampling. Amplification efficiency, in turn, is dictated by DNA template characteristics and the choice of a suitable gene marker and corresponding PCR primers (Piñol, Senar & Symondson, 2019). Factors such as DNA template quality, number of gene copies, genome size, taxonomic composition of the sample, primer biases resulting from the number, type, and position of template-primer mismatches, G+C content, length variation of amplicons among taxa, and the inherent trade-off between specificity and universality of primers can all alter amplification efficiency (Bellemain et al., 2010; Deagle et al., 2014; Geller et al., 2013; Stadhouders et al., 2010; Wintzingerode, Göbel & Stackebrandt, 1997). While it is impossible to design metabarcoding primers without biases, well-designed metabarcoding primers should be able to: (1) amplify a wide range of taxa (Clarke et al., 2017; Deagle et al., 2014; Zhan et al., 2014); (2) provide confident taxonomic identification at the highest possible resolution (Clarke et al., 2017; Deagle et al., 2014; Leray et al., 2013); (3) target a short barcode region that can be recovered from degraded DNA (Meusnier et al., 2008) and sequenced *via* high-throughput sequencing technologies (Engelbrektson et al., 2010; Huber et al., 2009); (4) differentiate between intraspecific and interspecific levels of genetic divergence (Čandek & Kuntner, 2015; Clarke et al., 2017; Coissac, Riaz & Puillandre, 2012; Deagle et al., 2014; Leray et al., 2013); and (5) minimize amplification of non-target DNA templates or bias among taxa (Elbrecht & Leese, 2015). The utility of a given primer set depends not only on primer-template amplification efficiency but also on the availability of curated and complete barcode databases for taxonomically verified voucher specimens (Casey et al., 2021; Cristescu, 2014; DiBattista et al., 2016; Gold et al., 2020; Ransome et al., 2017; Timmers et al., 2022).

The sessile and small-bodied organisms that account for the majority of biodiversity on coral reefs are understudied (Caldwell et al., 2024; Cowie, Bouchet & Fontaine, 2022), but molecular barcoding approaches provide valuable insights into this poorly described component of marine biodiversity (Leray & Knowlton, 2016; Nichols & Marko, 2019; Stat et al., 2017; Steyaert et al., 2022; Timmers et al., 2021; Timmers et al., 2022). Across most animal taxa, mitochondrial loci have the best level of variation to work broadly among taxa but also distinguish species (Hebert et al., 2003), leading to cyctochrome oxidase subunit I (COI) being the gold standard marker used to barcode the diversity of metazoans (Hebert & Gregory, 2005). Despite the success of COI across many marine benthic phyla, it performs poorly for some marine taxa that have very slow rates of mitochondrial sequence substitution, as seen in Anthozoans (Shearer et al., 2002), or fast rates, as observed in calcareous sponges (Lavrov et al., 2013). In particular, COI barcodes provide relatively little taxonomic resolution in phyla Porifera (sponges) and Cnidaria (jellyfish, anemones, corals, and their relatives), which include many of the most dominant and ecologically important taxa on coral reefs (Erpenbeck, Hooper & Worheide, 2006; Huang et al., 2008; Pöppe et al., 2010; Shearer et al., 2002). Currently, molecular surveys of coral reef biodiversity using DNA metabarcoding are hampered by the lack of primers and corresponding markers that capture a broad taxonomic scope of species across branches of the tree of life. Amplification primers that reliably detect and identify complex species-rich phyla in metabarcoding samples would likely aid with understanding ecological processes and greatly benefit our ability to monitor biodiversity on coral reefs in the face of climate change.

Here, we report new metabarcoding primers targeting a 318 bp fragment within the D1 region of the 28S rRNA nuclear gene. These novel primers detect a high taxonomic breadth of species across branches of the tree of life while being short enough for use on popular short-read high-throughput sequencing platforms. We compare the performance of this new primer pair to existing primers targeting the COI mitochondrial gene (Leray et al., 2013) in detecting the biodiversity of natural complex coral reef communities settled on Autonomous Reef Monitoring Structures (ARMS). Further, we select sponges (phylum Porifera) as a notoriously difficult taxonomic group on coral reefs to rigorously evaluate the performance of these novel primers in terms of species detection, amplification efficiency, and the quantitative potential for identifying coral reef taxa. To achieve this, we utilize ARMS as a means for a standardized comparison between metabarcoding and traditional imaged-based diversity estimates of coral reef communities (Casey et al., 2021; David et al., 2019; Obst et al., 2020; Vicente et al., 2022a; Vicente et al., 2022b), combined with artificial communities of known composition as a standardized way to recreate diverse ecological communities (Brown et al., 2015; Elbrecht & Leese, 2017; Govender et al., 2022; Krehenwinkel et al., 2017; Zhang et al., 2018).

Sponges are one of the most problematic of coral reef taxa for metabarcoding, because common markers lack species-level resolution for the majority of species within this group (Erpenbeck et al., 2016b; Erpenbeck et al., 2017; Schuster et al., 2018; Voigt, Eichmann & Wörheide, 2012a; Voigt, Wülfing & Wörheide, 2012b; Wörheide et al., 2002). In addition, sponge species are notoriously difficult to identify using either morphology or molecular taxonomy (Wörheide, Erpenbeck & Menke, 2007), explaining why they have received little attention relative to other reef-dwelling taxa (Fautin et al., 2010; GIGA Community of Scientists, 2014; Van Soest et al., 2012). However, they are critical components of coral reef ecosystems, with remarkable water filtering capacities (Gili & Coma, 1998) and host the largest diversity of microbial assemblages in the ocean (Thomas et al., 2016). Sponges are the only metazoans able to transform biologically unavailable forms of dissolved organic matter into nutritional, particulate organic carbon, providing resources for detritivores at lower trophic levels. This ‘sponge loop’ may fuel the great diversity of coral reef ecosystems (De Goeij et al., 2013; Rix et al., 2018). Given the high ecological relevance and the intrinsic challenges of detecting species within this group, sponges are important candidates to assess the performance of metabarcoding primers across the coral reef cryptobiome. Fortunately, an integrative taxonomic survey of the sponge cryptobiota has recently been completed for Kāneʻohe Bay (Vicente et al., 2022b) that provides both a comprehensive vouchered DNA sample and exhaustive 28S reference sequence database of every known species from this community. Using this as a resource, in addition to documenting performance of our new primers relative to COI, we also validate them extensively for sponges. This unique opportunity to combine complex natural coral reef samples for which we also have an exhaustive taxonomically validated and vouchered reference sequence library allows us to demonstrate the potential of these novel metabarcoding primers and highlights their utility to detect historically understudied cryptic coral reef phyla.

## Materials and Methods

### Primer design

We created an alignment of 29 species from 18 phyla across the tree of life, spanning the D1 region (Redmond et al., 2011) of the large subunit ribosomal RNA gene (hereby referred to as 28S rRNA) (Fig. S1). The sequences were downloaded from GenBank and include species within metazoans (phyla Cnidaria, Porifera, Bryozoa, Echinodermata, Annelida, Entoprocta, Mollusca, Platyhelminthes, Nemertea), algae (phyla Cercozoa, Streptophyta, Rhodophyta, Chlorophyta), bacteria (phyla Bacillariophyta, Cyanobacteriota, Pseudomonadota, Bacillota), and fungi (phyla Basidiomycota). We aligned the sequences using ClustalW in Geneious 10 (Kearse et al., 2012) with default parameters and manually inspected them to find the most suitable conserved regions targeting a ∼318 bp DNA fragment that would be compatible with high-throughput short-read sequencing technologies. Nucleotide degeneracy at a single base pair was incorporated in the design of the forward primer sequence to maximize amplification success across a wide range of coral reef taxa (Table 1).

**Table 1:** Sequence information of new 28S rRNA metabarcoding primer pair and COI primers used in this study.

Marker	Primer name	Sequence (5′-3′)	Direction	Reference
28S	jv28SD1F	TTCCCTCAGTAACGGCGAGY	F	This study
	LSU 300RV	CAACTTTCCCTCACGGTACTT	R	Redmond et al., 2011; Olson (NHM London, Per. Comm)
COI	mlCOIintF	GGWACWGGWTGAACWGTWTAYCCYCC	F	Leray et al., 2013
	jgHCO2198	TAIACYTCIGGRTGICCRAARAAYCA	R	Geller et al., 2013

**Notes.**

The IUPAC codes were used for degenerate nucleotides (Y = C + T; W = A + T; I = Isoleucine; R = A + G)

For validation using available references, a sponge (Phylum Porifera) specific alignment consisting of 243 sequences belonging to 241 species was generated using the same forward and reverse D1 region binding site of the novel 28S rRNA primer pair used for the 18 phyla (Text S1).

### Coral reef diversity—a comparison of 28S and COI primers performance in detecting biodiversity of homogenized bulk coral reef communities

**Figure 1:**
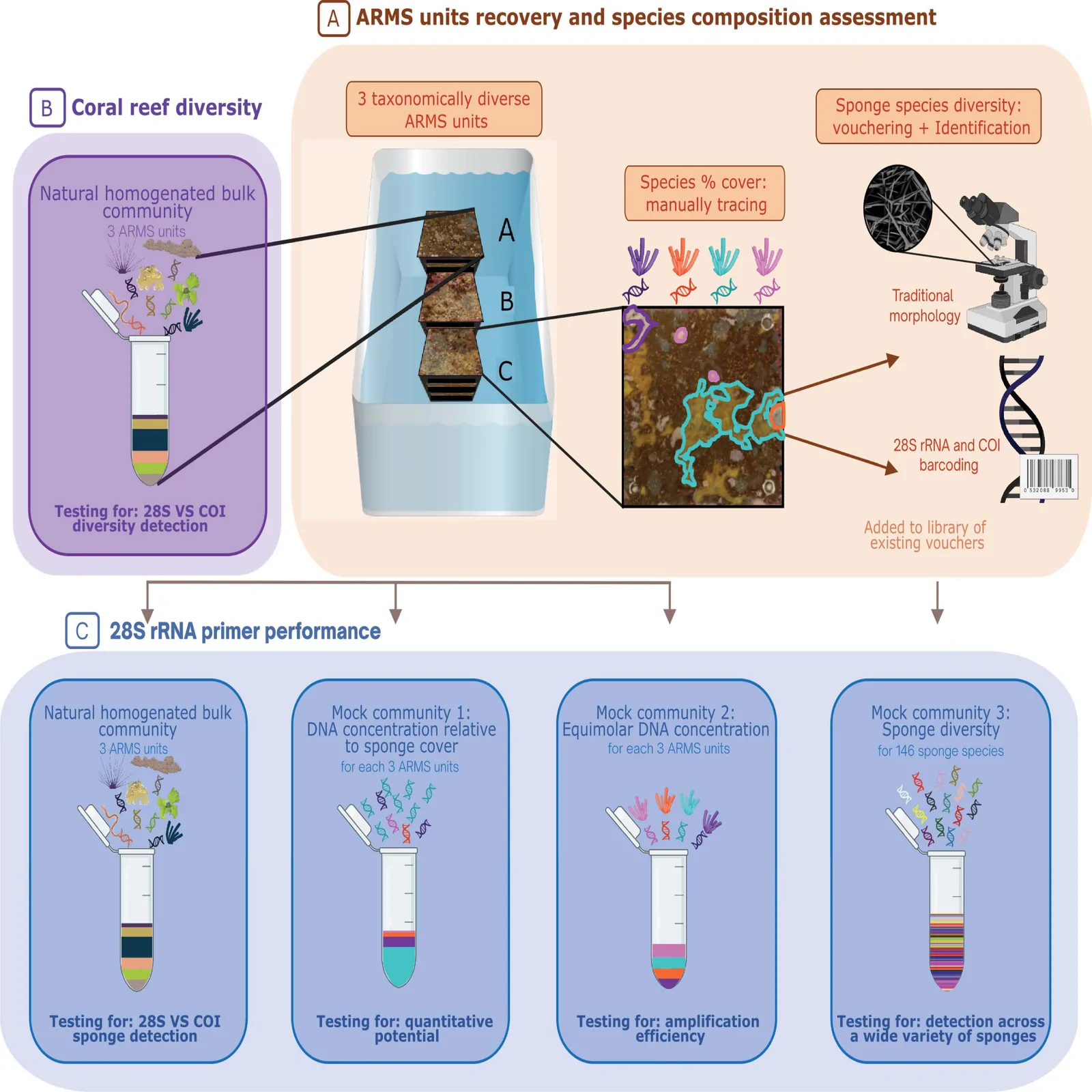
Overview of the experimental design. (A) Sponges were subsampled from three ARMS units as per (Timmers et al., 2022). The species’ taxonomic diversity from each ARMS unit was resolved using traditional morphology and molecular barcoding of unique species vouchered from the ARMS plates as per Vicente et al. (2022b) and the species abundance was measured by manually tracing the sponge surface area of each species on the plates. New vouchers were added to the existing library of Hawaiian sponges. (B) We compared the performance of our new 28S rRNA metabarcoding primers to COI (Leray et al., 2013; Geller et al., 2013; Table 1) for detecting species across different phyla of the tree of life on coral reef complex communities settled on the ARMS units. (C) We assessed the performance of novel 28S primer pair utilizing sponges as a target phylum on one natural and three artificial coral communities. We compare the detection performance and breadth of detection of sponges across markers (28S and COI) in the natural communities of the ARMS. We assembled three artificial mock communities of sponge species to determine the relationship between image-based percent cover and read abundance of species in DNA concentration mock community (mock community 1), primer bias across taxa (mock community 2), and recovery potential across a diverse library of sponges (mock community 3). Content for this figure was adapted from BioRender.com and the Integration and Application Network (ian.umces.edu/media-library).

To examine the performance of the novel 28S primers in detecting taxa across branches of the tree of life, metabarcoding was conducted on homogenized bulk extractions from complex coral reef communities that settled on three ARMS units (hereafter referred to as Unit A, B, and C) that were deployed and recovered in a mesocosm experiment conducted at the Hawaiʻi Institute of Marine Biology (HIMB) in Kāneʻohe Bay (see Timmers et al., 2021) for sampling and bulk extraction methods, Fig. 1A). To examine outcomes relative to other primers, we compared the performance of the 28S primers against the Leray-Geller COI primers (Leray et al., 2013; Geller et al., 2013; Table 1) the most commonly used marker in metabarcoding studies (Fig. 1B). The raw COI sequences for the same ARMS units were obtained from Timmers et al. (2021). We then went on to perform experimental validation of the primers using sponges as both a notoriously difficult group for metabarcoding and the best characterized taxon from these natural communities as outlined below.

### DNA metabarcoding

#### Library preparation

A touchdown PCR protocol was used to minimize non-specific amplification (Supplemental Information; Table 2). No template (negative) PCR controls were included throughout the library preparation process, and no contamination was detected by either agarose gel electrophoresis or DNA quantification (as detailed in the Text S1). Likewise, triplicate libraries of one ARMS unit (A) and its associated mock communities (three samples corresponding to nine libraries) were analyzed to quantify amplification variability within samples. Replicates were highly similar (Fig. S4), so we did not perform triplicate amplifications for the other samples. Post-amplification clean-up was accomplished with NGS Cleanup Magnetic Beads (Omega Bio-Tek). Ligation-based library preparation was conducted using either the NxSeq AmpFREE Low DNA Library Kit (Biosearch Technologies, Hoddesdon, UK) or the Kapa Hyper-Prep PCR-free Kit (Roche, Basel, Switzerland) (see Supplemental Information for details). Paired-end sequencing was performed on an Illumina MiSeq (v3 2x300PE) at the University of Hawaiʻi.

**Table 2:** Touchdown PCR protocol for newly-designed 28S rRNA metabarcoding primer pair.

Step	Phase	Temperature (°C)	Time
1	Initial denaturation	95	3 min
2	Denaturation	95	30 s
3	Annealing	50	30 s
4	Extension	72	1 min
Repeat steps 2–4 (3 cycles)			
5	Denaturation	95	30 s
6	Annealing	48	30 s
7	Extension	72	1 min
Repeat steps 5–7 (3 cycles)			
8	Denaturation	95	30 s
9	Annealing	45	30 s
10	Extension	72	1 min
Repeat steps 8–10 (40 cycles)			
11	Final extension	72	5 min
12	Final hold	4	∞

#### Sequence bioinformatics

Quality control, primer trimming, dereplication, merging of paired-end reads, and removal of chimeric sequences were performed using DADA2 (Callahan et al., 2016) on both 28S and COI datasets. Singletons were considered spurious sequences and were discarded in the detection analyses of species in all experiments. We rarefied the 28S and the COI datasets to equal sequencing depth for each ARMS community using the *Rarefy* command from the GUniFrac package in R (Chen et al., 2012). Resulting amplicon sequence variants (ASV) were annotated in two steps (Text S1) using: (1) the global GenBank nucleotide database and (2) a custom reference library encompassing all existing Hawaiian sponge vouchers with morphological descriptions, to control for the inaccuracy of public databases for sponges (Erpenbeck et al., 2016a; Wörheide & Erpenbeck, 2007). To generate this library, individual voucher specimens were amplified at both the 28S and COI loci whenever possible, and the resulting barcode sequences were included following Sanger sequencing.

### Primer performance—experimental validation of 28S primers on natural and mock communities of sponges

We conducted one natural community experiment and three mock community experiments to validate our novel 28S primers against the taxonomically most challenging group on coral reefs: Porifera.

#### Natural community: metabarcoding performance—a comparison of 28S and COI in detecting sponges

To compare the novel 28S primers’ ability to detect sponge taxa with the Leray-Geller COI primers, we used the same bulk genomic DNA extracts (for 28S) and raw sequences (for COI) of the three ARMS units as in the coral reef diversity comparison (‘Coral reef diversity–a comparison of 28S and COI primers performance in detecting biodiversity of homogenized bulk coral reef communities’). The species composition of the sponge community found on each ARMS was thoroughly assessed as described per Vicente et al. (2022b) (Fig. 1A), which allowed for a precise comparison of species and breadth of detections across both markers. A total of 29 species from 10 different orders were found across units (Table S1, Vicente et al., 2022b).

#### Mock community 1: quantitative potential—DNA concentration relative to sponge cover

To test the relationship between read abundance and sponge percent cover (a quantitative biomass proxy), we created artificial communities using DNA concentrations relative to the percent cover that each sponge occupied on the ARMS. We created a mock community for each of the three ARMS units. To achieve this, DNA extracts from each species found on the three individual ARMS were prepared (see Vicente et al., 2022b) for sponge sampling and extraction methods) (Fig. 1A) and were quantified using AccuBlue dsDNA broad range Quantitation Kit (Biotium, Fremont CA, USA) on a SpectraMax Microplate Reader (Molecular Devices, San Jose CA, USA). Sponges with an initial concentration lower than 10 ng/µL (∼27% of all sponges) were re-quantified using the AccuClear Ultra High Sensitivity dsDNA Quantitation Kit (Biotium, Fremont CA, USA) for greater accuracy. Photographs of recovered ARMS plates (top and bottom) were obtained as described per (Timmers et al., 2021). The surface area of each sponge across all plates within an ARMS unit was manually traced using ImageJ (Abràmoff, Magalhães & Ram, 2004; Fig. 1B). Surface area values were summed and converted into percent cover by dividing the summed values by the total potential surface area (3 plates × 2 sides of 22.9 length = 3,146.5 cm^2^ per ARMS unit). Concentrations directly proportional to the sponge species’ percent cover estimate calculated from the image software (Fig. 1C) were included. For example, if a species had 0.78% cover and another had 0.3% cover, the DNA concentrations included in the analysis would be of 10 ng/uL and 3.85 ng/uL, respectively. All sponge species present on the ARMS units were included except for Suberitidae sp. 3, which was removed from the percent coverage comparisons due to a labeling mistake that led to the inclusion of Suberitida sp. 3 in the mock communities (Table S1).

#### Mock community 2: community assemblage—sponge amplification efficiency across equimolar DNA concentration

To test for primer amplification bias among different sponges, we created a mock community for each ARMS unit by combining equimolar DNA concentrations of the unique sponge species observed within each unit (Fig. 1C). To obtain equimolar DNA concentrations, DNA extracts (as described for mock community 1) were normalized to 10 ng/µL if concentrations were >10 ng/µL or 1 ng/µL if the initial concentration was lower than 10 ng/µL. All 29 species were included (Table S1, Vicente et al., 2022b).

#### Mock community 3: sponge diversity—detection performance across a wide variety of sponges

To assess the detection potential of the newly designed primers across a wide diversity of sponges, we pooled equimolar DNA concentrations of 146 extracts from unique Hawaiian sponge species (Vicente et al., 2020; Vicente et al., 2022b; Bettcher et al., 2024; Vicente et al., 2025; Nunley et al., 2025; Fig. 1C, Table S2). Sponge extracts were prepared by collecting, vouchering, and extracting DNA from sponge samples collected in cryptic environments around patch reefs of Kāneʻohe Bay and from Autonomous Reef Monitoring Structure (ARMS), as described in Vicente et al. (2022b). The equimolar DNA concentration of sponges was obtained as described for mock community 2. This diverse assemblage of sponges included species from two orders within Calcarea (Clathrinida, Leucosolenida), 13 orders within Demospongiae (Axinellida, Biemnida, Chondrillida, Chondrosiida, Clionaida, Dendroceratida, Dictyoceratida, Haplosclerida, Poecilosclerida, Suberitida, Tethyida, Tetractinellida and Verongiida) and one order within Homoscleromorpha (Homosclerophorida) (Table S2).

### Statistical analyses

Data were analyzed using R version 4.1.0 (R Core Team, 2018), and visualizations were created using ggplot2 (Wickham, 2011).

To compare the taxonomic coverage of the 28S and COI primer sets across different coral reef phyla and classes, the number of unique species detected across the three ARMS units for the 28S and COI datasets was summed. The proportion of those species falling within different classes and phyla was calculated, and those results were visualized on a sunburst diagram. Species for which the phylum was unassigned were not considered for this analysis. We computed a two-sample *t*-test to assess the difference in species richness between 28S and COI metabarcoding within ARMS unit communities.

To evaluate the efficiency and accuracy of the new 28S primers for metabarcoding natural communities, the sponge species visually identified from ARMS units were compared with the metabarcoding results from the new 28S primers and existing COI primers. The presence/absence of sponge species across the three ARMS for both 28S and COI metabarcoding results were displayed on a heatmap. The overall detection success per marker was derived by dividing the total number of successful detections summed across the tree units by the number of expected detections based on visual surveys and was visualized on pie charts. To compare the taxonomic coverage of primer sets for Porifera, we calculated the average number of sponges detected by each 28S and COI metabarcoding per ARMS unit at the species, genus, family, order, and class levels and further compared those values to the actual community of the ARMS unit for each taxonomic rank.

We calculated the expected number of reads for each sponge within the different mock communities as follows: for mock community 1 (DNA concentration relative to percent coverage), the total number of reads per mock community was multiplied by the percent coverage of each sponge on the ARMS unit. For mock community experiment 2 (Equimolar DNA concentration), the total number of reads per community was divided by the number of sponges expected to be present in the mock community. Expected read abundances were calculated under the assumption that the primers amplify all sponge taxa with equal efficiency

Generalized linear models (GLM), as implemented in the Stats package, were used to inspect the relationships between initial percent cover based on images of ARMS plates and read number of sponges in both mock community 1 and natural community experiments. Given the overdispersion of the data, we opted for a quasi-Poisson distribution. We tested for a relationship between percent cover and read abundance of percent cover-based DNA concentration communities using the *cor.test* function implemented in the Stats package. We visually represented the relationship by a scatter plot on log_10_-transformed values for both variables using the *Ggscatter* function tool implemented in the Ggpubr package. To compare the performance of the 28S primers in conducting quantitative metabarcoding on sponge communities as opposed to complex natural coral reef communities, we computed the relative read abundance of sponges only within those communities. Log-transformed relative read abundance was visualized on a bubble chart along with the expected read abundance based on the percent coverage of sponges.

To assess the taxonomic biases of these primers, we calculated the fold deviation from the expected number of reads (observed–expected/ expected) and visualized those values on a bar chart for the sponges present in mock community 2. To test for statistical significance in the deviation between expected and observed read values, the Chi-Square deviation statistic for each sponge species was computed. To explore the underlying phylogenetic signal of primer biases, we generated a maximum likelihood topology in IQ-TREE (Nguyen et al., 2015) using ultrafast bootstrap approximation (1000 replicates) and ModelFinder (Kalyaanamoorthy et al., 2017) based on curated partial 28S rRNA sequences of sponges retrieved from GenBank.

To evaluate the detection breadth of the newly designed 28S primers, we calculated the percentage of sponges detected across the 146 known species included in mock community 3, presented by order.

## Results

### Primer design

The alignment of 29 species spanning a taxonomic scope of 18 distinct coral reef phyla revealed a highly conserved region across branches of the tree of life that we used to design our novel primer pair. This area shares >80% of the 20 base pairs within the binding site of each primer across diverse phyla including metazoans, algae, bacteria, and fungi (Figs. S1 and S5). 26 species have fewer than four mismatches at the primer binding site for each primer and three species (*Cytobacillus sponge*, *Amorphochlora amoeba*, and *Escherichia coli*) have had more than four mismatches. Within the sponges, there were no mismatches within the forward primer binding site for 166 species, whereas the reverse primer binding site matched the sequences of 239 out of the 241 sponge species (Figs. S2 and S3).

### Sequence bioinformatics of COI and 28S metabarcoding

Sequencing libraries yielded 5,418,300 paired-end reads (mean = 314,739 ± 240,877 standard deviation (SD)) for the 28S dataset and 492,708 demultiplexed paired-end reads (mean = 164,236 ± 22,346 SD) for the COI dataset. Following filtering, denoising, merging of paired-end reads, chimeric removal, and rarefaction, 1,004,792 sequences remained, corresponding to 1,482 ASVs for the 28S dataset, while the COI dataset yielded 268,179 sequences, corresponding to 1414 ASVs (see Table S5 for further details).

### Coral reef diversity—a comparison of 28S and COI primer performance in detecting biodiversity of homogenized coral reef bulk communities

**Figure 2:**
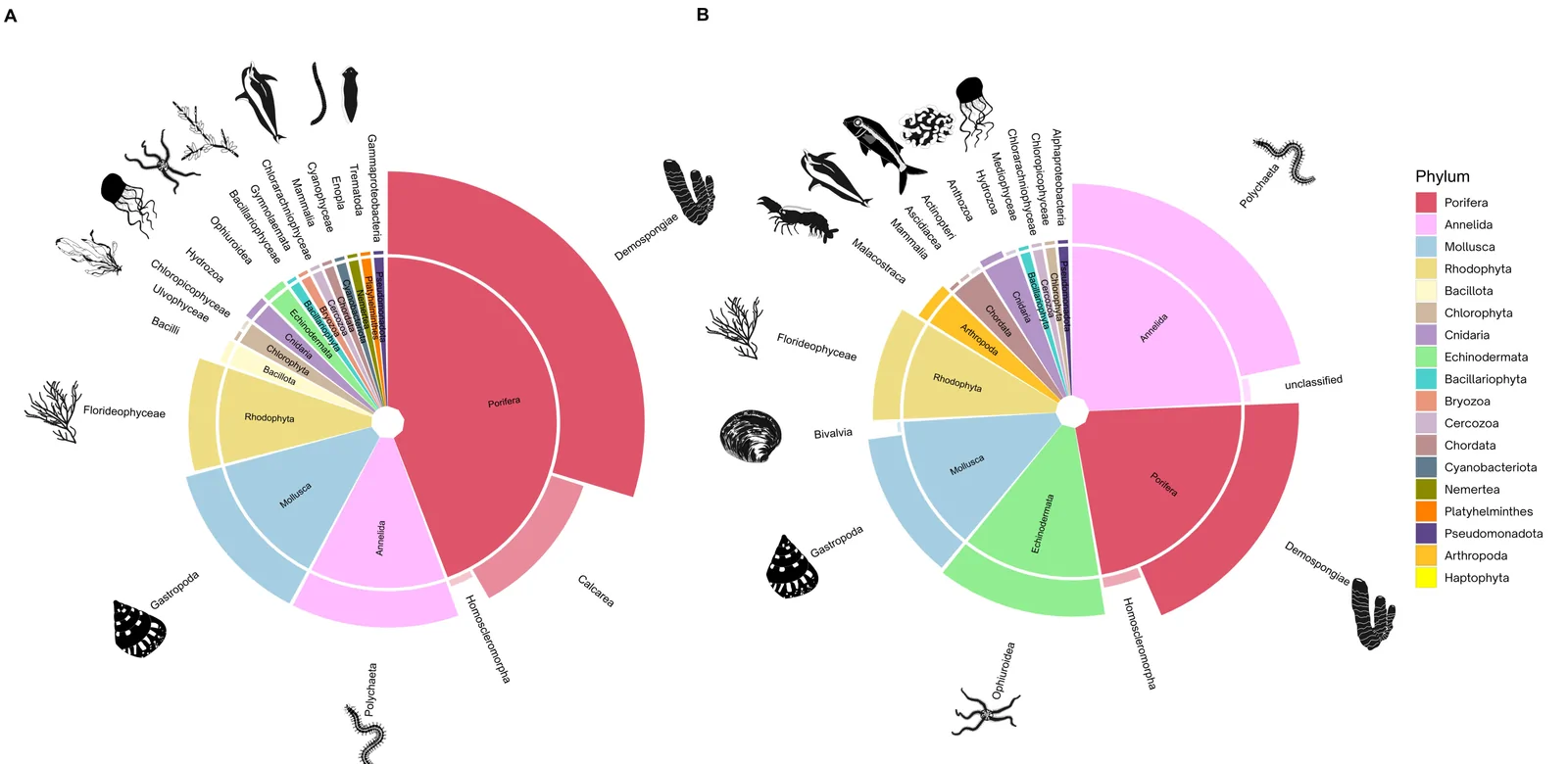
Comparison of the species taxonomic distribution within the complex reef community of ARMS across classes and phyla. We compared the taxonomic distribution using (A) the new 28S primer pair and (B) the COI primers. Colors depict different phyla, and level of shade shows different classes within a phylum. The length of the outer ring is proportional to the number of species present in each class. Each plot includes the total number of species summed across ARMS units A, B, and C. Content for this figure was adapted from BioRender.com and the Integration and Application Network (ian.umces.edu/media-library).

When comparing the ability of both primer pairs to detect species across branches of the tree of life, we found that the 28S primers captured species across 16 phyla encompassing 19 classes, while the COI primers successfully detected 18 classes within 12 phyla. For both primer sets, the majority of species detected were members of the phyla Porifera, Annelida, Mollusca, and Rhodophyta (Fig. 2). However, the number of species detected within each phylum and respective classes differed across markers. Most notably, the 28S primers detected more species within the phyla Porifera (*n* = 37) than the COI primers (*n* = 19). Both markers performed equally in detecting species within Rhodophyta (*n* = 8) and Mollusca (*n* = 11). In contrast, the COI primers recovered a greater taxonomic scope within the phyla Annelida (*n* = 20), Cnidaria (*n* = 3), and Echinodermata (*n* = 11) compared to the 28S primers (*n* = 11, 2 and, 2, respectively). At the class level, the results for both primers were similar within each of these major phyla, except for Porifera, Cnidaria, and Mollusca. Among Porifera, COI primers detected classes Demospongiae and Homoscleromorpha but failed to detect any species within the sponge class Calcarea (see primer validation below for more details). In contrast, the 28S primers missed the Cnidarian class Anthozoa but detected a higher number of species than the COI primers within the class Hydrozoa. The COI primers also detected species within the class Bivalvia, but that group was not represented in the 28S data set. Among the less speciose groups, the 28S primers detected five phyla that were absent from the COI data set (Bryozoa, Cyanobacteriota, Platyhelminthes, Nemertea, and Bacillota), whereas COI detected one phyla (Arthropoda) that was undetected by 28S metabarcoding. Despite those differences in the taxonomic distribution of species diversity across branches of the tree of life, the per-unit species richness observed did not differ significantly between markers (*t* (3.65) = −0.177, *ρ* = 0.158, Table S6).

### Primer performance—experimental validation of 28S primers on natural and mock communities of sponges

#### Natural community: metabarcoding performance—a comparison of 28S and COI in detecting sponges

**Figure 3:**
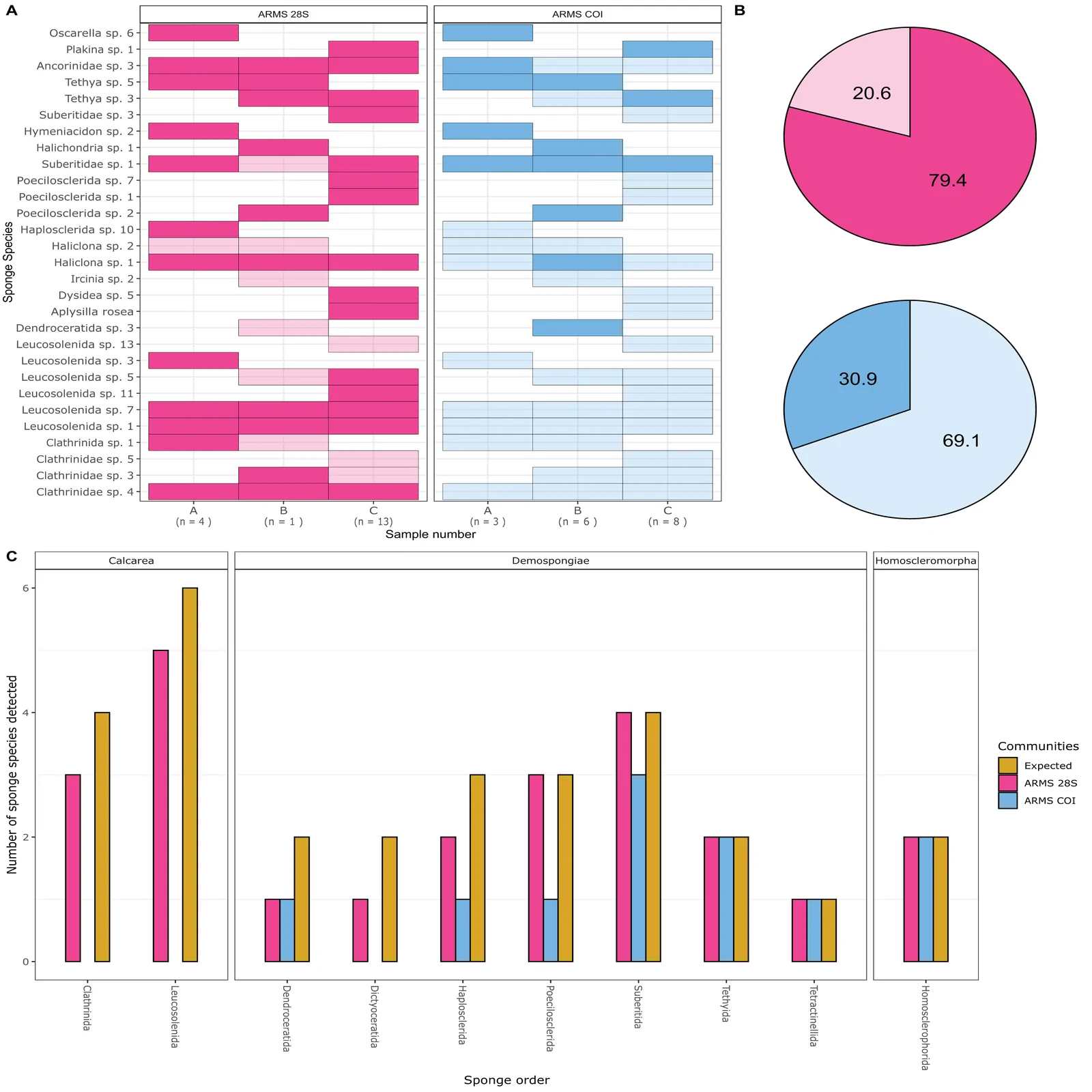
Comparison of metabarcoding efficiency between the new 28S primer pairs and COI primers in Leray et al., (2013). (A) Heatmap of detection (presence/absence) of the 29 sponge species (*n* = 47 individuals) present on the three ARMS units communities by the COI (right) and the 28S (left) primers. The darker shaded colors represent successfully detected species confirmed through integrative taxonomy, whereas lighter colors represent species that were missed by the primers but confirmed by integrative taxonomy. The numbers at the bottom of the heatmap represent the number of false positives (incorrectly detected sponge species) in each community. (B) The pie charts represent the percentage of the sponge community that was successfully captured by the 28S (top) and COI (bottom) primers. (C) Bar chart of the number of species detected for each of the orders represented in the ARMS mock communities. The number of taxa detected with 28S and COI primers is represented by the pink and blue bands, respectively. The expected number of taxa at each phylogenetic level was estimated from visual annotations and taxonomic surveys of ARMS units (represented by yellow bars). The orders are grouped into classes on the *x*-axis and labeled at the top.

In the natural reef community experiment, COI primers detected only 30.9% of sponge species present in the ARMS (Fig. 3B). In contrast, species detection increased to 79.4% when using the newly designed 28S primer pair (Fig. 3B). There was a ∼2.3-fold difference in successfully captured species detections between the two primer sets (*n* = 14 for COI *vs* 37 for 28S: Fig. 3A).

The 28S primers detected 92% of species within ARMS A, 63% in ARMS B, and 83% within ARMS C. In contrast, the COI marker captured only 38% of species in ARMS A, 38% in ARMS B, and 17% in ARMS C. The 28S primers detected at least 50% (up to 100%) of the species within each order, compared to the COI primers, which failed to detect a single species in the orders Clathrinida, Leucosolenida, and Dicyoceratida (Fig. 3C). The 28S primers recovered the majority of species across all orders present in the mock community (*n* = 10), whereas the COI primers consistently detected fewer (or no) species in six out of the ten orders sampled. Specifically, the 28S primers outperformed the COI primers in detecting species across the orders Clathrinida, Leucosolenida, Dictyoceratida, Haplosclerida, Poecilosclerida and Suberitida, whereas the two primer pairs performed equally in detecting species within the orders Dendroceratida, Tethyida, Tetractinellida and Homoscleromorpha (Fig. 3C). The COI marker failed to capture any species belonging to the class Calcarea, a common taxon in the field samples and throughout the 28S dataset. Further, the 28S primers outperformed the COI primer set when detecting sponge taxa at every taxonomic level (species, genus, family, order, and class) across ARMS unit communities (Fig. S6). Despite this improvement, ∼17% (*n* = 8) of species were still missed across both marker data sets; these missed taxa belonged to the orders Clathrinida, Leucosolenida, Haplosclerida, and Dictyoceratida. In terms of overlap, the COI primers detected one species that was missed by the 28S primers, whereas 15 species detected by the 28S primers were missed by COI metabarcoding.

Similar rates of false-positive sponge species that were erroneously detected in the ARMS communities due to sequencing errors or species misidentification were found between the two primers. Although present in low abundance, false positives accounted for ∼27%, on average, of sponge detections per community with the COI marker, whereas an average of ∼28% of sponge species detected per ARMS using the 28S primers were false positives not detected during visual surveys (Table S7).

#### Mock community 1: quantitative potential—DNA concentration relative to sponge cover

**Figure 4:**
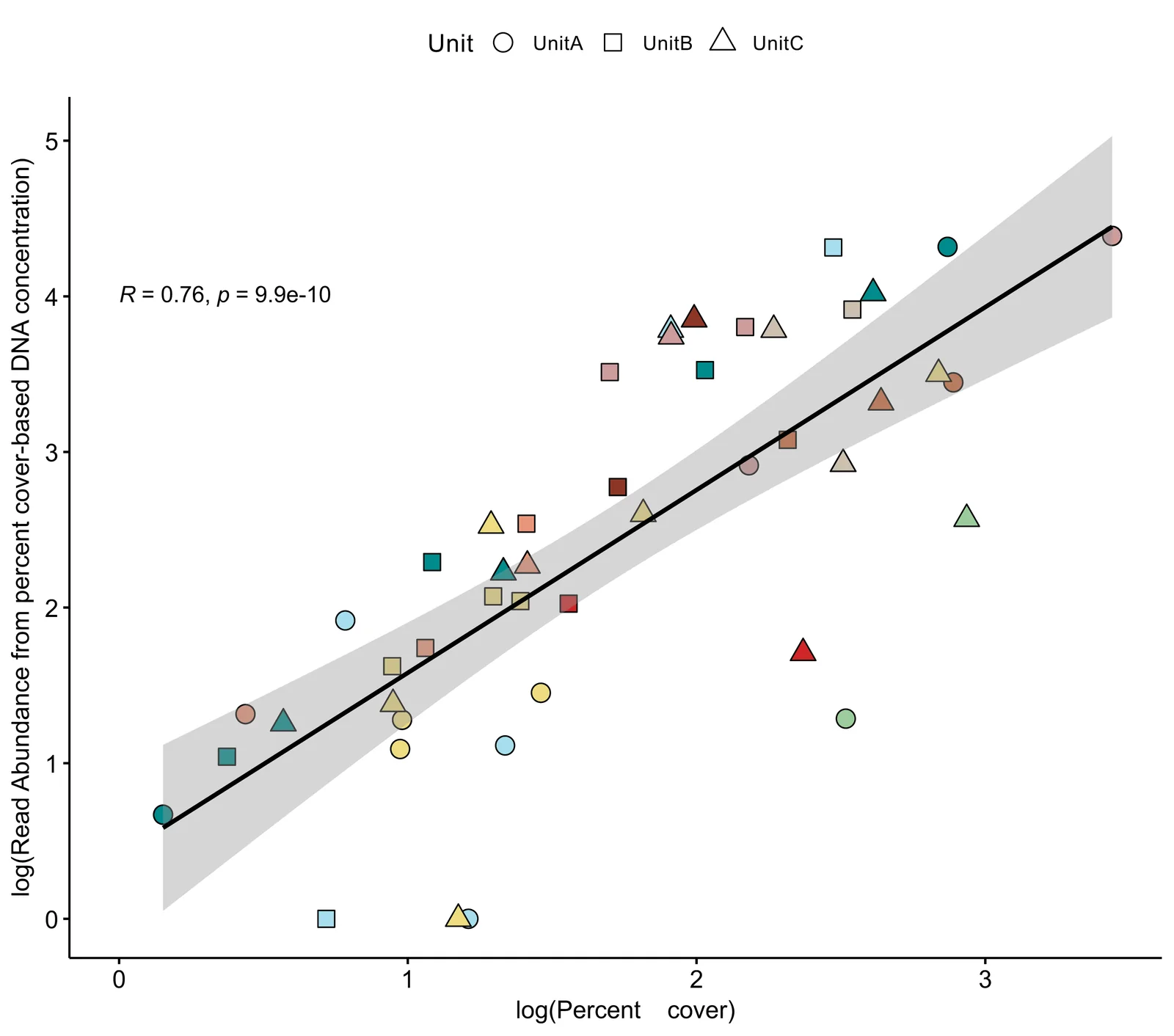
Relationship between the percent cover and the read abundance of sponges. Analysis was done with the percent cover-based DNA concentration mock communities (mock community 1), expressed on a log scale. The three mock communities contain a total of 28 sponge species. Read abundance was obtained by sequencing three assembled mock communities with initial DNA concentration proportional to percent cover. Percent cover was obtained from manually tracing communities on ARMS plates with ImageJ. A linear regression and 95% confidence intervals are plotted, indicating a significant log-linear relationship between species percent cover and read abundance (*R* = 0.76, *ρ* = 9.9 e-10). Colors depict different sponge orders.

Three of the 46 sponges present in mock community 1 were undetected by the 28S primers (a species within the order Leucosolenida and *Haliclona* sp. 2). We found a significant and positive linear correlation (*R* = 0.76, *ρ* < 0.01) between the percent cover of image traced sponges with their read abundances (Fig. 4). Although there is variation among species, relative abundance of sponges (from % cover) is generally predicted by read number in the metabarcoding libraries (GLM *ρ* < 0.01, Table S8).

Further, qualitative representation of relative read abundance of species was similar between the percent cover-based DNA concentration mock communities, expected values, and the ARMS units communities (Fig. S7). Presence of false negatives and positives had a greater occurrence in the metabarcoding analysis of the ARMS with recruitment of the natural coral reef community than in the mock communities made from percent cover-based-DNA concentrations. However, the read abundance of species that were successfully detected on ARMS units matched the expected values (GLM, *ρ* = 0.0136, Table S9).

#### Mock community 2: community assemblage—amplification efficiency across equimolar DNA concentration

**Figure 5:**
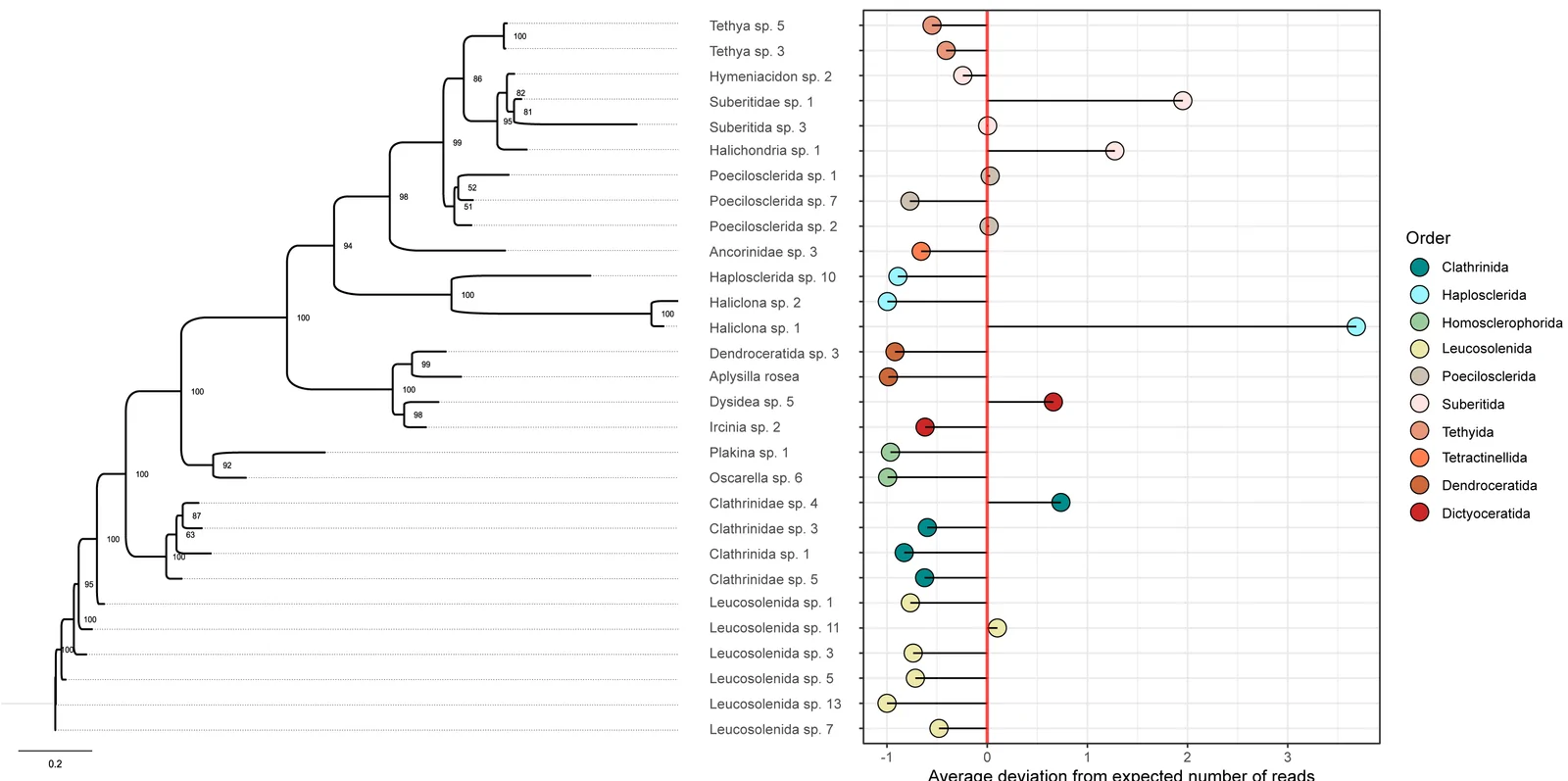
Detection performance of the 28S primers on 29 sponge species present within three equimolar mock communities (mock community 2). The red line represents the average number of reads expected for each species based on initial DNA concentration without amplification bias. The fold deviation from expected number of reads (observed –expected/expected) was computed for each sponge and averaged across the 3 mock communities (A, B and C) and are represented as circles. Maximum likelihood topology (left) was generated in IQtree from curated partial 28S rRNA gene sequences retrieved from GenBank. Bootstrap values are indicated at each node.

28S primers successfully recovered 97% of sponge species (28/29) contained in mock community 2 (equimolar DNA concentration) used to test the primer for amplification biases. Despite relative read abundance matching mock community input reasonably well, observed read abundance among sponges differed significantly from the expected uniform read number (Tables S10–S12: *ρ* < 0.01 for each mock community, Fig. 5). Some species-specific biases were responsible for the significant deviation. Notably, the sequence abundance for *Haliclona* sp. 1 was roughly three-fold higher than expected. With the exception of the orders Tethyida and Homoscleromorpha, the average deviation from expected read abundance varied among species within orders, with species displaying negative and positive deviations from the expected read number. Overall, the average deviation from expected read abundance was similar across orders (+/- one-fold, Table S13), so there are no clear phylogenetic patterns underlying primer amplification biases, as there are with COI.

#### Mock community 3: sponge diversity—detection performance across a wide variety of sponges

**Figure 6:**
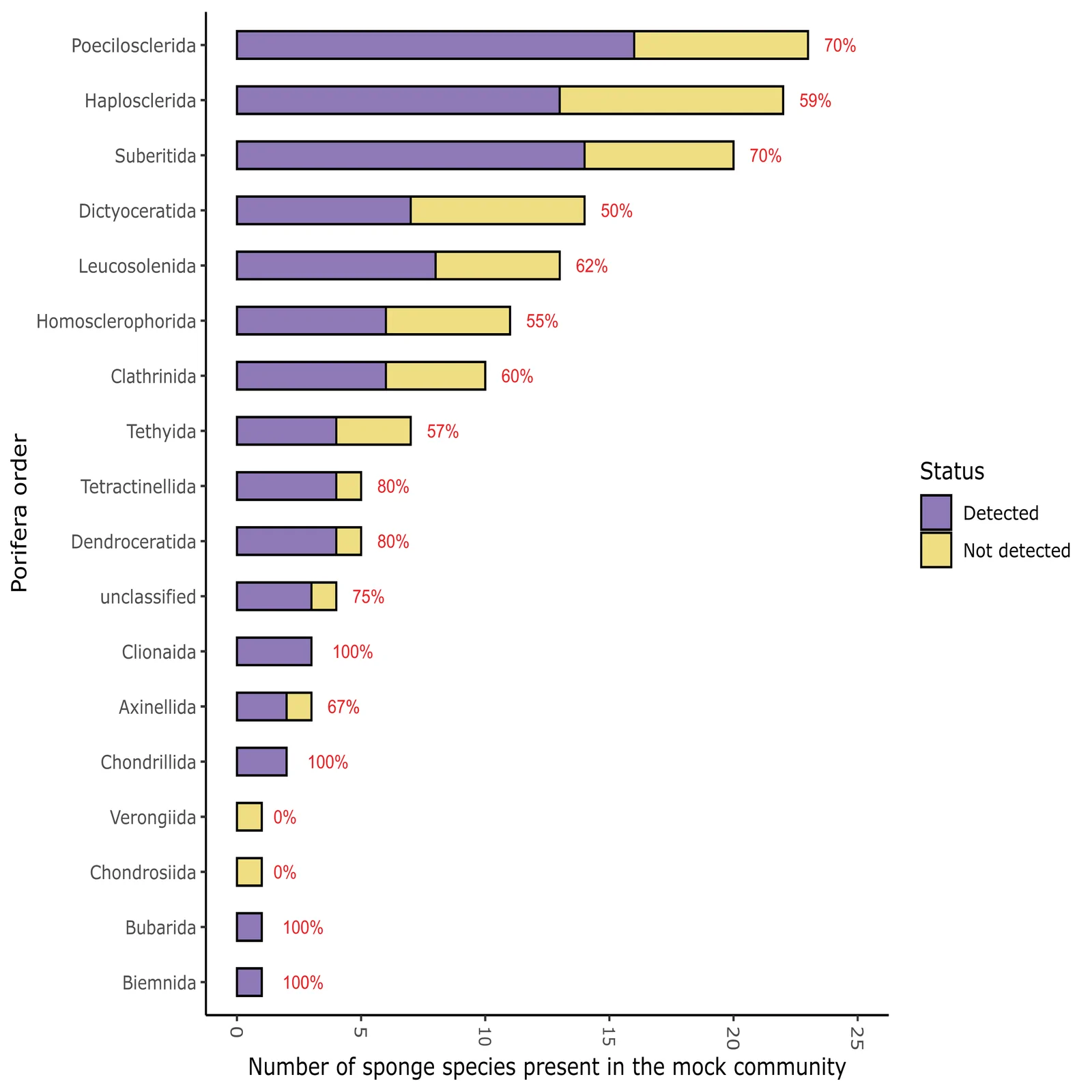
Detection performance of 28S primers across a highly diverse mock community of 146 sponge species (mock community 3). The yellow bands indicate species that were not detected with the primers, whereas the purple bands indicate species detected by the primers. Percentage of species successfully detected within each order is indicated in red.

Nearly 65% (94/146) of sponge species present in the mock community were successfully recovered using the 28S primer (Fig. 6). Detections spanned 17 orders (two orders within Calcarea, 14 orders within Demospongiae, and one order within Homoscleromorpha in addition to four species unclassified at the order level). Species detection success rate was higher than 50% for 15 of the 17 orders. The primers performed best at detecting species within the orders Tetractinellida (80%), Dendroceratida (80%), Clionaida (100%), Chondrillida (100%), Bubarida (100%), and Biemnida (100%). The only sponges missed by the primers were among the rarest in the mock community (Chondrosiida and Verongiida), although Biemnida and Bubarida also had among the fewest species and still showed a 100% detection rate.

## Discussion

Coral reefs are the most diverse and species-rich marine habitats and are also among the most severely threatened ecosystems on the planet (Carpenter et al., 2008; Hoegh-Guldberg et al., 2007). With a majority of coral reef biodiversity yet to be formally described (Fautin et al., 2010; Fisher et al., 2015; Mora et al., 2011), there is a pressing need to document and conserve coral reef biodiversity that is potentially being lost before we can even discover it (Scheffers et al., 2012; Sebens, 1994). Given the cost associated with biodiversity monitoring in remote and understudied areas, many are turning to molecular methods as affordable and scalable alternatives to traditional monitoring techniques (DiBattista et al., 2019; Elbrecht & Leese, 2015; Leray & Knowlton, 2015; Stat et al., 2017). However, the accuracy and comparability of studies based on metabarcoding-derived molecular assessments of biodiversity depend on understanding the accuracy, detection limits, and variability among taxa and primers (De Barba et al., 2014; Hatzenbuhler et al., 2017; Lamb et al., 2019; Stat et al., 2017). Here, we design a novel primer pair and introduce a novel metabarcoding marker targeting the D1 region of the 28S gene. We found this region is highly conserved across functionally important groups on coral reefs including metazoans, algae, bacteria, and fungi. We used natural coral reef communities that settled on ARMS units to demonstrate the broad application of our novel primer pair across coral reef biodiversity and compared its detection performance with the most used marker to survey coral reef biodiversity (COI). We show that our novel primers uncovered a greater taxonomic breadth than the COI primers across coral reef benthic phyla and classes, although each shows some possible taxonomic biases in terms of taxa that were missed.

We demonstrated the potential of our novel primer pair to detect a broad taxonomic scope of species spanning 18 phyla across several kingdoms of the tree of life (metazoans, bacteria, fungi, and algae). By design, our novel primers can capture the plethora of diversity found within a complex coral reef ecosystem, and despite a more depauperate reference sequence database for the D1 region, these primers perform equal to or better than existing metabarcoding primer resources. To demonstrate this, we use COI as the most used marker for metazoan metabarcoding on coral reefs and compare its species distribution across phyla and classes to data obtained with our novel 28S primers. We found that the 28S primers uncovered a broader taxonomic scope of classes and phyla than the COI primers on natural complex coral reef communities that settled on ARMS, despite no difference in species richness. Because we lack a taxonomic survey of species (other than sponges, below) against which to validate our results, we cannot draw precise conclusions about the taxonomic accuracy of the species detection using both primer pairs. The differences in species detection of metazoans and prokaryotes across primers reveal limitations and taxonomic biases that should be carefully considered when selecting a primer set for metabarcoding surveys. Despite known biases and limitations, the COI primers have been adopted as a standard target to uncover coral reef metazoan diversity through molecular surveys due to their ability to detect species across several key coral reef phyla, such as Porifera, Annelida, Mollusca, Rhodophyta, Cnidaria, Echinodermata, and others. We demonstrate that our novel 28S primer pair can achieve similar results with an even broader taxonomic scope of detection, despite fewer references in public databases, making them promising candidates for molecular biodiversity assessments of complex coral reef communities. Since we did not use a manually curated database to detect non-sponge metazoans, we cannot tease apart whether the lack of specificity of both primers in detecting metazoan species is caused by actual detection failure or incomplete and inaccurate public barcode databases. However, the COI is the most widely utilized barcode across the entire animal kingdom (Hebert & Gregory, 2005), which underscores the extensiveness of public databases for this marker in contrast to other less common markers like the 28S, which ought to be disadvantaged in this regard. Additional thorough investigations of the 28S and COI primer performance across non-sponge phyla in complex sessile and motile coral reef communities of known composition could benefit our understanding of the biases and potential complementarity of those primers across branches of the tree of life. Harnessing the strengths of each marker towards a multi-locus approach to survey the diversity of highly diverse coral reef communities would be an interesting avenue to explore.

We selected sponges as the most taxonomically challenging coral reef phyla to perform an in-depth validation of the performance of these novel metabarcoding primers because we also have an exhaustive, manually curated reference sequence database of all the known species in this group derived from a recent taxonomic survey of the area (Vicente et al., 2022b). Using a combination of artificial mock communities from these vouchers and natural communities of known species composition and abundance, we show that the 28S primers performed significantly better across a larger taxonomic breadth than existing COI primers. These novel primers amplified most species within ∼two-fold relative to the initial DNA concentration and can provide a rank order estimate of individual species abundance within natural communities. Despite missing some species, our novel primer pair successfully detected most sponge species with read counts proportional to their biomass across a wide variety of taxa in artificial and natural species-rich communities. The fact that some evolutionarily distinct species of coral reef sponges bear very similar sequences, whereas others that are thought to be closely-related show high genetic differentiation (Erpenbeck et al., 2016b) highlights the intrinsic challenge of designing metabarcoding primers for broad biodiversity surveys that can pick up this species-rich and understudied group. For that reason, we selected sponges as one of the most challenging taxonomic groups on coral reefs to perform an in-depth assessment of these novel 28S primers’ performance and achieve a thorough comparison with existing COI primers. We demonstrated that our novel primers recovered most of the species diversity in the ARMS unit communities and outperformed the mainstream metabarcoding COI primers for detecting sponge species in complex coral reef communities. For example, the 28S primers successfully recovered a class of sponges (Calcarea) that was entirely missed by COI and that, despite its ubiquity, has not been represented in metabarcoding studies targeting benthic metazoans on coral reefs. The 28S primers also had broader taxonomic coverage, detecting a greater number of species per sponge order compared to the COI primers and identifying more species across each taxonomic rank (species, genus, family, order, and class). It is relevant to note that the higher number of PCR cycles used to amplify samples with the 28S primers compared to the COI primer (>40x and 36x, respectively) could have influenced the detection of rare species in metabarcoding libraries (Witzke et al., 2020).

Sequencing of ARMS communities with both markers yielded some false negatives of species that were missed by metabarcoding regardless of marker choice. Overall, failed detections of sponges that were known to be present were much more common for COI than 28S. All species present in the natural and mock communities were individually barcoded with the 28S primers but only half of those succeeded with the COI primers using Sanger sequencing of a taxonomic voucher before being added to a manually curated database against which we conducted taxonomic assignments for both markers. The disparity in success rates between 28S and COI barcoding efforts for all vouchers does suggest that COI reference database incompleteness compared to 28S databases is a factor that explains detection improvement of the 28S metabarcoding primer. However, in the case of Calcarea (23 operational taxonomic units (OTUs)) it is impractical to complete the COI reference database since their mitochondrial genomes are fragmented into multiple linear chromosomes (Lavrov & Gettle, 2026). Moreover, as mitochondrial genomes continue to be sequenced, we will be able to eliminate OTUs for which database completeness could be ruled out and focus on factors such as primer biases that prevent the detection of all species and stochasticity of the PCR reaction to potentially explain the detection failure of some sponges using the 28S and COI primers.

It is noteworthy that the sequencing of ARMS communities with both markers also resulted in some false-positives, sponge species that were not included in the mock communities but detected by the metabarcoding (Table S7). Overall, false detections of sponges that we could not find during taxonomic identification were roughly equal between the two metabarcoding primers. These false positives may have been missed in visual surveys, but more likely originate from errors in public reference databases that contain misidentifications. Relatively few sponge barcodes in GenBank are anchored to a taxonomically verified voucher specimen so it is difficult to assess the frequency of misidentification, but it is believed to be very high among the sponge taxonomic community. The difficulties in sponge classification at most taxonomic levels, combined with the declining levels of taxonomic expertise, have resulted in notoriously incomplete and inaccurate barcode libraries for sponges (Erpenbeck et al., 2016b; Wörheide & Erpenbeck, 2007). Further, neither primer pair could detect genetic differences between closely related species in a handful of cases. On the other hand, factors such as sequencing errors, tag-jumping, or microscopic sponges on the ARMS plates that are missed during visual assessments could also create putative false positives in our sequence library. We find that the detection of species we could not observe visually was substantially higher among naturally occurring communities. We do not know if this is because we amplified sponge recruits that could not yet be identified visually or because we include other taxa that complicate the analyses. However, the fact that the false positive rate is so similar between primer pairs (27% *vs* 28%) suggests that neither marker has a real advantage in this regard.

Given the novel 28S primers’ demonstrated potential for detecting sponges with high species resolution across a broad taxonomic scope in natural coral reef communities, we chose to explore how these primers can be applied for quantitative metabarcoding. We included a mock community with DNA concentration of sponges relative to their coverage calculated on image-based analysis of natural communities settled on the ARMS and found that species coverage of sponges in complex coral reef communities of ARMS units was correlated with the read abundance of 28S amplicons. However, we got a more accurate estimate of read-based abundance when only sponges were present (mock community 1) rather than when sponges were mixed with other metazoans on ARMS units (Fig. S7). We found that sponge species with the lowest percent coverage on the ARMS plates were primarily responsible for that discrepancy in abundance estimates’ success. The presence of numerically dominant species on ARMS plates could have hindered the detection of specimens with very low template amounts (Deagle et al., 2014; Leray & Knowlton, 2017). Previous studies also find that specimens with low abundance are more often missed by metabarcoding and have variable sequence read numbers (Elbrecht & Leese, 2015). However, other factors, such as primer amplification efficiency, could also explain some observed variations in species detection and read abundance.

To test this, we explored the amplification efficiency of the 28S primers using a mock community of the same sponges present at equimolar DNA concentrations. We found that primer amplification efficiency was relatively consistent across sponge species and orders in our equimolar mock community. However, we did observe significant species-specific biases for *Haliclona* sp. 1 (order Haplosclerida), as well as for the orders Homosclerophorida (*Plakina* sp. 1 and *Oscarella* sp. 6) and Tethyida (*Tethya* sp. 3 and *Tethya* sp. 5). Factors such as G+C content or length of amplicons could contribute to the preferential amplification of species such as *Haliclona* sp. 1, but even in this case observed read abundance was only ∼3-fold higher than expected from input DNA concentration. Although significant, any such biases must be small for final DNA concentrations to fall within 1-3 fold, because a primer efficiency difference of just 1% would result in orders of magnitude difference in DNA concentration across 40 amplification cycles. Alternatively, template-primer mismatches can cause some taxa to amplify less successfully than others; a single base pair mismatch at the primer binding site can yield a 1000-fold decrease in read abundance (Clarke et al., 2014). Such stochasticity and primer biases may play a role in the species we failed to detect within our mock communities, either because of mismatches or low DNA quality or quantity relative to our expectations. Finally, pipetting error and PCR stochasticity are likely responsible for some of the observed variation, and could easily explain 1-3 fold differences in final read abundance. For example, Clathrinidae sp. 4 and Suberitidae sp. 1 displayed both positive and negative deviations from expected read numbers in different mock communities (Table S13), indicating that stochastic effects drive some of the observed variations.

Despite potential biases, the 28S amplicon read abundance predicts species abundance within about ∼two-fold. Thus, within that level of uncertainty, relative abundance metrics for these species can be inferred from metabarcoding read numbers, consistent with previous studies (Aylagas et al., 2016; Hänfling et al., 2016; Leray & Knowlton, 2015; Nichols & Marko, 2019; Ushio et al., 2017). The emerging consensus is that metabarcoding datasets provide robust assessments of complex ecological communities and can be leveraged as tools for environmental monitoring and to guide policymaking. However, it is relevant to note that the ARMS and mock communities we analyzed in this study had low species evenness (*i.e.,* the abundance of each species in the mix is highly dissimilar), which tends to be optimal for performing rank-order abundance estimates with metabarcoding (Piñol, Senar & Symondson, 2019).

Finally, we demonstrated the broad application of our novel 28S primer pair across the phylum Porifera and showed that it successfully detected the majority of sponges in our diverse and species-rich mock community 3. While they captured a broad range of taxa, the primers still failed to pick up some sponge species. Factors such as stochasticity of the PCR reaction, primer biases, DNA template quality or quantity, and amplicon length could potentially explain some of the detection failures. It is important to note that multiple taxonomic species still had identical or very similar 28S sequences, indicating that this small fragment within the D1 region of the 28S lacks the resolution to successfully resolve all sponges to the species level. This was particularly evident in the orders Suberitida, Clathrinida, and Leucosolenida and the genera *Tethya* and *Haliclona* (Table S4), for which the resolution power of the marker would be more suitable for taxonomic assignments at the genus and family levels. For example, this lack of resolution likely explains why Leucosolenida sp. 13 was not detected because the sequence within the D1 region of the 28S was exactly the same as the sequence of Leucosolenida sp. 7, and both were present in the same mock community. Thus, only one of these “species” could be reliably identified. Regardless, our novel 28S shows great promise for detecting previously overlooked species among highly-diverse cryptic coral reef communities.

## Concluding remarks

We report a new metabarcoding marker targeting the D1 region of the 28S rRNA gene and demonstrate its ability to detect equal or greater coral reef diversity relative to the most commonly used Leray-Geller COI metabarcoding primers. We show that our novel primers amplify a higher taxonomic scope of species across many key cryptic coral reef phyla than COI primers and are thus promising candidates for surveys of coral reef biodiversity, either alone or in combination with COI. While COI-based methodologies developed to date (*e.g.*, Elbrecht & Leese, 2017; Geller et al., 2013; Leray et al., 2013) are the bedrock of metabarcoding studies and will undoubtedly continue to provide important insights, combining this novel primer with COI markers may be a pragmatic solution to offset the trade-off between taxonomic breadth and species resolution in metabarcoding studies of metazoans (Deagle et al., 2014). We further validated the performance of these novel primers in the most taxonomically challenging group on coral reefs (sponges) using a combination of natural and artificial mock communities with imaged-based visual censuses of ARMS units and an exhaustive manually curated reference sequence database. These ribosomal markers detect greater taxonomic breadth and resolution of sponge species than COI. Despite some taxon-specific amplification failures, we found that these new primers have low amplification biases, capture a broad range of taxa in species-rich communities, and show potential to predict relative abundances of species in artificial and natural communities. Our study highlights the value of validating metabarcoding primers against standard biodiversity census techniques to ensure that primers can successfully assess the species composition of complex ecological assemblages. We recommend using a standardized framework such as artificial mock communities plus natural complex coral reef communities of known composition (such as ARMS units) to validate metabarcoding performance and better understand results from complex environmental samples.

## Supplemental Information

10.7717/peerj.21662/supp-1 Supplemental Information 1 Supplemental text, figures, and tables

## Additional Information and Declarations

### Data Availability

The following information was supplied regarding data availability: The data and code are available at GitHub and Zenodo: - https://github.com/martineauG/28S_marker_paper
- martineauG. (2026). martineauG/28S_marker_paper: First Stable Release (Version v1.0.0) [Computer software]. Zenodo. https://doi.org/10.5281/zenodo.20737134
The raw sequence data are available at GenBank: PRJNA1149044, SRR30275729–SRR30275738, SRR30316297–SRR30316304, SRR30316420–SRR30316422, MW016190, PQ317792, PQ317793, MW016051, PQ317794, MW016104, PQ317795, PQ317796, MW016142, MW016168, PQ317797, PQ317798, MW016227, PQ317799, PQ317800, PQ317801, MW016275, PQ317802, MW016290, PQ317803, PQ317805, MW016364, PQ317806, PQ317807, PQ317808, PQ317809, PQ317810.
