# Supplementary material for: Introducing a novel 28S rRNA marker for improved assessment of coral reef biodiversity
Source: PeerJ. 2026 Aug 24;14:e21662. doi: 10.7717/peerj.21662 (PMC13525748; doi:10.7717/peerj.21662)
Supplement: Supplementary file 1 [file peerj-14-21662-s001.docx]

Supplementary Material for

**Introducing a novel 28S rRNA marker for improved assessment of coral reef biodiversity**

Gabrielle Martineau, Robert J. Toonen, Molly A. Timmers, Christopher P. Jury, Jan Vicente

**Table of Contents**

[Supplementary Text	4](#_Toc198448774)

[Primer design and alignment	4](#_Toc198448775)

[Supplementary Text	5](#_Toc198448776)

[Quality filtering of Next Generation Sequence data using DADA2	6](#_Toc198448777)

[Supplementary Text	7](#_Toc198448778)

[Taxonomic Assignment of Sponges	7](#_Toc198448779)

[Figure S1	8](#_Toc198448780)

[Alignment of 29 species spanning 18 different phyla in the D1 region of the 28S rRNA gene targeted by the novel 28S primers presented in this study.	8](#_Toc198448781)

[Figure S2	9](#_Toc198448782)

[Alignment of 166 sequences belonging to 164 sponge species against the novel 28S forward primer jv28SD1F	9](#_Toc198448783)

[Figure S3	12](#_Toc198448784)

[Alignment of 241 sponge species against the reverse 28S primer	12](#_Toc198448785)

[Figure S4	15](#_Toc198448786)

[The species taxonomic composition of replicates from bulk scrapings of naturally settled sponges on ARMS Unit A (left panel), manually created equimolar mock communities of sponges identified on ARMS unit A (mock community 2) (center panel) and mock communities based on the percent cover of sponges from image analysis of ARMS unit A (mock community 3) (right panel) sequenced with the 28S primers.	15](#_Toc198448787)

[Figure S5	16](#_Toc198448788)

[Alignment of the forward and reverse novel 28S rRNA primer pair binding sites against 29 species from 18 phyla	16](#_Toc198448789)

[Figure S6	17](#_Toc198448790)

[Taxonomic coverage comparison of sponge species detection at different taxonomic levels using different markers on communities associated with three ARMS units. The number of taxa detected with the 28S and COI primers are represented by the pink and blue bands, respectively. The expected number of taxa at each phylogenetic level was calculated from visual annotations of ARMS units and are represented by the yellow bands.	17](#_Toc231465875)

[Figure S7	18](#_Toc198448792)

[Bubble chart comparing read relative abundance of species in 1) percent cover-based DNA concentration mock communities (mock community 3) ARMS units communities, and 3) expected values based on traced percent coverage for units A, B, and C	18](#_Toc198448793)

[Table S1	19](#_Toc198448794)

[Composition (taxonomic diversity and percent coverage) of mock communities 2 (equimolar DNA concentrations) and 3 (DNA concentration relative to percent coverage) for each ARMS unit	19](#_Toc198448795)

[Table S2	20](#_Toc198448796)

[Sponge species present in mock community 1 (detection performance across a wide variety of sponges)	20](#_Toc198448797)

[Table S3	24](#_Toc198448798)

[Additional sponge 28S rRNA barcodes deposited in GenBank. Taxonomic classification and GenBank accession numbers are provided.	24](#_Toc198448799)

[Table S4	26](#_Toc198448800)

[Taxonomic identification swaps made in a custom R script, prioritizing the species known to be present in mock communities 1, 2, and 3 and in the ARMS unit. Those swaps were made when 1) species were tied for the top BLAST hit or when 2) the species identified in the top BLAST hit was a close relative to a species known to be present in the mix.	26](#_Toc198448801)

Table S5	28

[Read abundance at different steps of the bioinformatics pipeline for all samples sequenced.	28](#_Toc198448803)

[Table S6	29](#_Toc198448804)

[Two-sample t-test comparison of unit-by-unit species richness between COI and 28S metabarcoding of complex coral reef communities on ARMS units.	29](#_Toc198448805)

[Table S7	30](#_Toc198448806)

[Read abundance of false positive sponge species detections in ARMS units communities for 28S and COI datasets.	30](#_Toc198448807)

[Table S8	31](#_Toc198448808)

[Generalized linear model of the relationship between image-based percent cover and read abundance of species in percent cover-based DNA concentration mock community 3.	31](#_Toc198448809)

[Table S9	32](#_Toc198448810)

[Generalized linear model of the relationship between image-based percent cover and read abundance of species in ARMS units communities.	32](#_Toc198448811)

[Table S10	33](#_Toc198448812)

[Chi-square test of expected and observed read abundance for each sponge species for ARMS A	33](#_Toc198448813)

[Table S11	34](#_Toc198448814)

[Chi-square test of expected and observed read abundance for each sponge species for ARMS B.	34](#_Toc198448815)

[Table S12	35](#_Toc198448816)

[Chi-square test of expected and observed read abundance for each sponge species for ARMS C.	35](#_Toc198448817)

[Table S13	36](#_Toc198448818)

[Fold deviation from the expected number of reads for the Equimolar cocktail.	36](#_Toc198448819)

# Supplementary Text

## *Primer design and alignment*

The forward primer (jv28SD1F) was aligned with 166 sequences belonging to 164 species (Figure S2 and the reverse primer (LSU 300RV) was aligned with sequences belonging to 241 species Figure S3 All sequences included representatives of 63 genera within 19 orders from the three dominant sponge classes (Calcarea, Demospongiae, and Homoscleromorpha). Of these, 146 are from Hawaiian sponge species, and the remaining 95 sponge species sequences were downloaded from Genbank to increase taxonomic breadth for marker generality. The 146 Hawaiian sponge species were previously identified through an integrated taxonomic survey (Vicente et al. 2020; 2022b) that includes all known species from the local community, although many of them remain to be formally described. All sponge tissue samples are vouchered at the Hawaiʻi Institute of Marine Biology, Bernice Pauahi Bishop Museum and the Florida Museum of Natural History. Currently, only 13 of these 146 species have been formally described and 10 of those are new to science (Bettcher et al., 2024; Nunley et al., 2025; Vicente et al., 2025). Sequences downloaded from GenBank were limited to individual sponge species published in peered review articles that were curated and identified by taxonomic experts, including 50 from Thacker et al. (2013), 23 from Voigt et al. (2012), 10 from Gazave et al. (2010; 2013), and 3 from Melis et al. (2016) and Idan et al. (2018).

# Supplementary Text

## *Library preparation*

PCR amplifications occurred in 20µL total reaction volume comprising 0.6 µL of each primer (10 µM), 10 µL of GoTaq Green 2X Master Mix (Promega, Madison WI, USA), 0.15 µL of BSA (1 mg/mL), 7.65 µL of nanopure H_2_O and 1 µL of template DNA. The success of PCR amplifications (defined as the presence of a single band ~ 318 bp) was verified on 2% agarose gel stained with GelRed (Biotium, Freemont, CA, USA).

For both kits, end-repair and a-tailing reactions were performed following the manufacturer's protocol. For the NxSeq libraries, we ligated Illumina sequencing adapters (Illumina, San Diego CA, USA) to amplicons following the manufacturer protocol. For the Kapa Hyper-Prep, XGen Stubby Adapters (Integrated DNA Technologies, Coralville IA, USA) were ligated to cleaned PCR products (15 minutes at 20°C), followed by a post-ligation bead clean-up (1:0.8 amplicons: beads). Unique Dual Index (UDI) combinations (Integrated DNA Technologies) were then ligated to all Kapa libraries using the profile: 95℃ for 45 s; 10 cycles of 98℃ for 15 s, 60℃ for 30 s, and 72℃ for 30 s; and 72℃ for 1 min. The indexed PCR products from NxSeq and Kapa libraries were purified using a 1X ratio of NGS Cleanup Magnetic Beads, and eluted in 30 μL of molecular grade water.

All libraries were quantified using AccuBlue dsDNA broad range Quantitation Kit (Biotium, Freemont, CA, USA) on a SpectraMax Microplate Reader (Molecular Devices, San Jose, CA, USA) following manufacturer protocols, visually inspected on a 2% agarose gel, and quality validated using a Bioanalyzer chip (Agilent Technologies, Santa Clara CA, USA). We performed paired-end sequencing of the normalized libraries on two runs of an Illumina MiSeq (v3 2x300PE) at the University of Hawaiʻi.

# Supplementary Text

## *Quality filtering of Next Generation Sequence data using DADA2*

Sample demultiplexing was performed on COI raw reads obtained from Timmers et al., 2021 using Cutadapt 4.9 (Martin et al., 2011), allowing no errors in indices matching. Quality control, primer trimming, dereplication, merging of paired-end reads, and removal of chimeric sequences were performed using DADA2 (Callahan et al., 2016) on both 28S and COI datasets. Briefly, the *filterAndTrim* command was applied for primer trimming (*trimLeft* argument) and quality filtering (forward and reverse truncation lengths of 260 and 180 for 28S and 280 and 280 for COI, respectively; forward and reverse maximum expected error of 2 and 4 for both markers). We used the *learnErrors* and *derepFastq* commands for error rate learning and dereplication, respectively. Paired-end reads were merged using the *mergePairs* command, and the *DADA* command was used to infer amplicon sequence variants (ASV). We screened the reads for chimeras using the *removeBimeraDenovo* command.

# Supplementary Text

## *Taxonomic Assignment of Sponges*

Uncurated public databases are notoriously inaccurate for sponges (Erpenbeck, Ekins, et al., 2016; Wörheide & Erpenbeck, 2007). To overcome this limitation, we created a reference sequence library encompassing all known taxonomic vouchers for Hawaiian sponges with morphological descriptions, including all the sponge species present in the mock communities (n_species_ = 146) for which a barcode could be generated. This curated reference library contained 291 sequences representing 153 unique taxa (28S) and 108 sequences representing 76 unique taxa (COI) in previous studies (Vicente et al., 2022a; Vicente et al., 2020). 27 of these 291 28S sequences are novel barcodes generated in this study for species who’s existing 28S fragments in GenBank were missing and did not overlap with our primer region (Table S3). Our inability to acquire COI barcodes for those species who’s 28S sequences were generated was due to the unreliability of the COI marker to amplify all sponge taxa (Timmers et al., 2021). Taxonomic assignments were performed in two steps to allow for 1) the precise identification of sponge taxa with our curated sponge reference library and 2) the identification of non-sponge taxa found in the homogenated bulk coral reef communities of the ARMS using the global GenBank nucleotide database. Accordingly, amplicons were queried against a local sponge database (BLASTn) created from our reference library with the parameters of E value < 1e-3, minimum query coverage of > 95%, and minimum percent identity of 95%. Taxonomy was assigned based on the match with the highest sequence similarity to queries using our curated local database. If several identities were tied for the highest match, a custom R script prioritizing sponges known to be present in mock communities was used to assign taxonomy (Table S4). To identify non-sponge ASVs, we subsequently annotated the sequences that remained taxonomically unassigned using BLASTn matches against the entire GenBank nucleotide database with the same parameters and downstream filtering criteria. We eliminated sequences for which the best match was below 95% identity in downstream analyses.

# Figure S1

Alignment of 29 species spanning 18 different phyla in the D1 region of the 28S rRNA gene targeted by the novel 28S primers presented in this study.

# Figure S2

## Alignment of 166 sequences belonging to 164 sponge species against the novel 28S forward primer jv28SD1F

# Figure S2 continued

# Figure S2 continued

# Figure S3

Alignment of 241 sponge species against the reverse 28S primer LSU 300RV

# Figure S3 continued

# Figure S3 continued

# Figure S4

The species taxonomic composition of replicates from bulk scrapings of naturally settled sponges on ARMS Unit A (left panel), manually created equimolar mock communities of sponges identified on ARMS unit A (mock community 2) (center panel) and mock communities based on the percent cover of sponges from image analysis of ARMS unit A (mock community 3) (right panel) sequenced with the 28S primers. We calculated read relative abundance for each species, sample, and replicates. The 19 most abundant species are displayed, whereas the less common species were grouped together as “Other”. Different colors represent the different species. Species highlighted in bold in the legend are sponges.

# Figure S5

## Alignment of the forward and reverse novel 28S rRNA primer pair binding sites against 29 species from 18 phyla

# Figure S6

## Taxonomic coverage comparison of sponge species detection at different taxonomic levels using different markers on communities associated with three ARMS units. The number of taxa detected with the 28S and COI primers are represented by the pink and blue bands, respectively. The expected number of taxa at each phylogenetic level was calculated from visual annotations of ARMS units and are represented by the yellow bands.

# Figure S7

Bubble chart comparing read relative abundance of species in 1) percent cover-based DNA concentration mock communities (mock community 3) ARMS units communities, and 3) expected values based on traced percent coverage for units A, B, and C. The size of bubbles is proportional to the log_10_ of the read relative abundance. The different colors represent different orders of Porifera.

| Table S1  Composition (taxonomic diversity and percent coverage) of mock communities 2 (equimolar DNA concentrations) and 3 (DNA concentration relative to percent coverage) for each ARMS unit | | | | | | |
| --- | --- | --- | --- | --- | --- | --- |
| Sponge class | Sponge order | Sponge species | 28s rRNA GenBank accession number | Unit percent coverage (A) | Unit percent coverage (B) | Unit percent coverage (C) |
| Calcarea | Clathrinida | Clathrinida sp. 1 | MW016082 | 0.0009 | 0.0031 | - |
|  |  | Clathrinidae sp. 5 | MW016097 | - | - | 0.0190 |
|  |  | Clathrinidae sp. 3 | MW016092 | - | 0.0249 | 0.0060 |
|  |  | Clathrinidae sp. 4 | MW016095 | 1.6211 | 0.2370 | 0.0453 |
|  | Leucosolenida | Leucosolenida sp. 1 | MW016193 | 0.0187 | 0.0526 | 1.5279 |
|  |  | Leucosolenida sp. 3 | MW016216 | 0.0184 | - | - |
|  |  | Leucosolenida sp. 5 | MW016218 | - | 0.0175 | 0.0175 |
|  |  | Leucosolendia sp. 7 | MW016223 | 0.0611 | 0.0419 | 0.1429 |
|  |  | Leucosolendia sp. 11 | MW016197 | - | - | 0.0409 |
|  |  | Leucosolendia sp. 13 | MW016202 | - | - | 0.0309 |
| Demospongiae | Dendroceratida | *Aplysilla rosea* | MW016050 | - | - | 0.5171 |
|  |  | Dendroceratida sp. 3 | MW016107 | - | 0.0784 | - |
|  | Dictyoceratida | *Dysidea* sp. 5 | MW016120 | - | - | 0.2156 |
|  |  | *Ircinia* sp. 2 | MW016185 | - | 0.1170 | - |
|  | Haplosclerida | *Haliclona* sp. 1 | MW016135 | 0.0111 | 0.6632 | 0.1785 |
|  |  | *Haliclona* sp. 2 | MW016139 | 0.0333 | 0.0094 | - |
|  |  | Haplosclerida sp. 10 | MW016152 | 0.0454 | - | - |
|  | Poecilosclerida | Poecilosclerida sp. 1 | MW016274 | - | - | 0.7123 |
|  |  | Poecilosclerida sp. 2 | MW016275 | - | 0.7729 | - |
|  |  | Poecilosclerida sp. 7 | MW016292 | - | - | 0.4087 |
|  | Suberitida | *Halichondria* sp. 1 | MW016126 | - | 0.3273 | - |
|  |  | *Hymeniacidon* sp. 2 | MW016175 | 6.0303 | - | - |
|  |  | Suberitidae sp. 1 | MW016299 | 0.3307 | 0.1097 | 0.1791 |
|  |  | Suberitida sp. 3 | MW016325 | - | - | - |
|  | Tethyida | *Tethya* sp. 3 | MW016339 | - | 0.0553 | 0.0553 |
|  |  | *Tethya* sp. 5 | MW016344 | 0.0038 | 0.0234 | - |
|  | Tetractinellida | Ancorinidae sp. 3 | MW016040 | 1.6976 | 0.4602 | 0.9644 |
| Homoscleromorpha | Homosclerophorida | *Oscarella* sp. 6 | MW016259 | 0.7189 | - | - |
|  |  | *Plakina* sp. 1 | MW016264 | - | - | 1.9125 |
| *Note.* Percent cover refers to the surface area of each sponge species in reference to the entire ARMS unit area. Sponge species absent from a mock community are denoted by a “-.” Due to a labeling mistake, that led to the inclusion of Suberitida sp. 3 instead of Suberitidae sp. 3 in mock communities 1, 2, and 3, that species was not included in mock community 3 for percent cover analyses. | | | | | | |

| Table S2  Sponge species present in mock community 1 (detection performance across a wide variety of sponges) | | | |
| --- | --- | --- | --- |
| Sponge class | Sponge order | Sponge species | 28s rRNA GenBank accession number |
| Calcarea | Clathrinida | Clathrinida sp. 1 | MW016082 |
|  |  | Clathrinidae sp. 2 | MW016090 |
|  |  | Clathrinidae sp. 3 | MW016092 |
|  |  | Clathrinidae sp. 4 | MW016095 |
|  |  | Clathrinidae sp. 5 | MW016097 |
|  |  | Clathrinidae sp. 8 | MW016098 |
|  |  | Clathrinidae sp. 9 | MW016099 |
|  |  | Clathrinidae sp. 10 | MW016087 |
|  |  | Clathrinidae sp. 11 | MW016088 |
|  |  | Leucettidae sp. 3 | MW016190 |
|  | Leucosolenida | Leucosolenida sp. 1 | MW016193 |
|  |  | Leucosolenida sp. 2 | MW016209 |
|  |  | Leucosolenida sp. 3 | MW016216 |
|  |  | Leucosolenida sp. 5 | MW016218 |
|  |  | Leucosolendia sp. 7 | MW016223 |
|  |  | Leucosolenida sp. 9 | MW016226 |
|  |  | Leucosolenida sp. 10 | MW016196 |
|  |  | Leucosolenida sp. 11 | MW016197 |
|  |  | Leucosolenida sp. 12 | MW016200 |
|  |  | Leucosolenida sp. 13 | MW016202 |
|  |  | Leucosolenida sp. 19 | MW016208 |
|  |  | Leucosolenida sp. 20 | MW016212 |
|  |  | Leucosolenida sp. 21 | MW016214 |
|  | unclassified | Calcinea sp. 1 | MW016056 |
| Demospongiae | Axinellida | Axinellida sp. 1 | MW016051 |
|  |  | *Echinodictyum* sp. 1 | MW016122 |
|  |  | Raspailiidae sp. 2 | MW016311 |
|  | Biemnida | *Biemna fistulosa* | MW016054 |
|  | Bubarida | Bubarida sp. 1 | MW016358 |
|  | Chondrillida | Chondrillida sp. 1 | MW016074 |
|  |  | Chondrillida sp. 3 | MW016075 |
|  | Chondrosiida | Chondrosiida sp. 1 | MW016076 |
|  | Clionaida | Clionaida sp. 2 | MW016365 |
|  |  | *Spirastrella sp. 1* | MW016318 |
|  |  | *Spheciospongia solida* | MW016316 |
|  | Dendroceratida | *Aplysilla rosea* | MW016050 |
|  |  | *Chelonaplysilla erecta* | MW016067 |
|  |  | *Chelonaplysilla* sp. 1 | MW016069 |
|  |  | *Chelonaplysilla* sp. 3 | MW016070 |
|  |  | Dendroceratida sp. 1 | MW016104 |
|  |  | Dendroceratida sp. 3 | MW016107 |
|  | Dictyoceratida | Dictyoceratida sp. 2 | MW016108 |
|  |  | Dictyoceratida sp. 3 | MW016109 |
|  |  | Dictyoceratida sp. 4 | MW016110 |
|  |  | Dictyoceratida sp. 5 | MW016111 |
|  |  | Dictyoceratida sp. 6 | MW016112 |
|  |  | *Dysidea cf. arenaria* | MW016115 |
|  |  | *Dysidea cf. pallescens* | MW016116 |
|  |  | *Dysidea* sp. 3 | MW016118 |
|  |  | *Dysidea* sp. 5 | MW016120 |
|  |  | *Dysidea* sp. 6 | MT452546 |
|  |  | *Dysidea* sp. 9 | MW016121 |
|  |  | Dysideidae sp. 1 | MW016368 |
|  |  | *Ircinia* sp. 2 | MW016185 |
|  |  | *Lamellodysidea cf. chlorea* | MW016189 |
|  | Haplosclerida | *Callyspongia* sp. 1 | MW016060 |
|  |  | *Callyspongia* sp. 2 | MW016375 |
|  |  | Chalinidae sp. 2 | MW016065 |
|  |  | *Cladocroce* sp. 1 | MW016079 |
|  |  | *Gelliodes wilsoni* | MW016123 |
|  |  | *Haliclona* sp. 1 | MW016135 |
|  |  | *Haliclona* sp. 2 | MW016139 |
|  |  | *Haliclona* sp. 3 | MW016145 |
|  |  | *Haliclona* sp. 21 | MW016140 |
|  |  | *Haliclona* sp. 23 | MW016142 |
|  |  | *Haliclona* sp. 24 | MW016143 |
|  |  | Haplosclerida sp. 1 | MW016149 |
|  |  | Haplosclerida sp. 2 | MW016161 |
|  |  | Haplosclerida sp. 3 | MW016165 |
|  |  | Haplosclerida sp. 6 | MW016166 |
|  |  | Haplosclerida sp. 7 | MW016167 |
|  |  | Haplosclerida sp. 8 | MW016168 |
|  |  | Haplosclerida sp. 10 | MW016152 |
|  |  | Haplosclerida sp. 12 | MW016154 |
|  |  | Haplosclerida sp. 14 | MW016159 |
|  |  | Haplosclerida sp. 15 | MW016361 |
|  | Poecilosclerida | *Monanchora clathrata* | MW016229 |
|  |  | *Iotrochota protea* | MW016181 |
|  |  | *Lissodendoryx (Waldoschmittia) hawaiiana* | MT452537 |
|  |  | *Lissodendoryx* sp. 1 | MW016227 |
|  |  | *Lissodendoryx* sp. 2 | MW016228 |
|  |  | *Mycale* sp. 9 | MW016241 |
|  |  | *Mycale* sp. 10 | MW016233 |
|  |  | *Mycale* sp. 14 | MW016236 |
|  |  | *Mycale* sp. 15 | MW016237 |
|  |  | *Mycale (Zigomycale) parishii* | MW016231 |
|  |  | Poecilosclerida sp. 1 | MW016274 |
|  |  | Poecilosclerida sp. 2 | MW016275 |
|  |  | Poecilosclerida sp. 3 | MW016285 |
|  |  | Poecilosclerida sp. 4 | MW016286 |
|  |  | Poecilosclerida sp. 6 | MW016290 |
|  |  | Poecilosclerida sp. 7 | MW016292 |
|  |  | Poecilosclerida sp. 9 | MW016295 |
|  |  | Poecilosclerida sp. 10 | MW016277 |
|  |  | Poecilosclerida sp. 11 | MW016279 |
|  |  | Poecilosclerida sp. 12 | MW016280 |
|  |  | *Tedania cf. klausi* | MW016326 |
|  |  | *Tedania* sp. 1 | MW016329 |
|  | Suberitida | *Halichondria coerulea* | MW016125 |
|  |  | *Halichondria* sp. 1 | MW016126 |
|  |  | *Halichondria* sp. 2 | MW016128 |
|  |  | *Halichondria* sp. 4 | MW016131 |
|  |  | *Halichondria* sp. 5 | MW016132 |
|  |  | Heteroscleromorpha sp. 9 | MW016313 |
|  |  | *Hymeniacidon* sp. 1 | MW016173 |
|  |  | *Hymeniacidon* sp. 2 | MW016175 |
|  |  | *Hymeniacidon* sp. 3 | MW016178 |
|  |  | *Hymeniacidon* sp. 5 | MW016180 |
|  |  | *Suberites* sp. 1 | MW016320 |
|  |  | Suberitida sp. 1 | MW016321 |
|  |  | Suberitida sp. 2 | MW016322 |
|  |  | Suberitida sp. 3 | MW016324 |
|  |  | Suberitida sp. 4 | MW016364 |
|  |  | Suberitidae sp. 1 | MW016299 |
|  |  | Suberitidae sp. 2 | MW016302 |
|  |  | Suberitida sp. 3 | MW016325 |
|  |  | Suberitidae sp. 4 | MW016307 |
|  |  | Suberitidae sp. 5 | MW016308 |
|  |  | Suberitidae sp. 6 | MW016309 |
|  |  | *Terpios* sp. 1 | MW016331 |
|  | Tethyida | *Tethya* sp. 1 | MT452533 |
|  |  | *Tethya* sp. 2 | MW016337 |
|  |  | *Tethya* sp. 3 | MW016339 |
|  |  | *Tethya* sp. 4 | MW016341 |
|  |  | *Tethya* sp. 5 | MW016344 |
|  |  | *Tethya* sp. 6 | MW016347 |
|  |  | Tethyidae sp. 1 | MW016351 |
|  | Tetractinellida | Ancorinidae sp. 1 | MW016374 |
|  |  | Ancorinidae sp. 2 | MW016037 |
|  |  | Ancorinidae sp. 3 | MW016040 |
|  |  | Ancorinidae sp. 4 | MW016041 |
|  |  | Ancorinidae sp. 5 | MW016042 |
|  |  | Ancorinidae sp. 7 | MW016048 |
|  |  | Ancorinidae sp. 8 | MW016049 |
|  | unclassified | Heteroscleromorpha sp. 3 | MW016169 |
|  |  | Keratosa sp. 1 | MW016187 |
|  |  | Keratosa sp. 2 | MW016188 |
|  |  | Verongimorpha sp. 1 | MW016352 |
|  | Verongiida | *Pseudoceratina sp. 1* | MW016310 |
| Homoscleromorpha | Homosclerophorida | *Corticium sp. 1* | MW016103 |
|  |  | *Oscarella* sp. 1 | MW016246 |
|  |  | *Oscarella* sp. 2 | MW016249 |
|  |  | *Oscarella* sp. 3 | MW016251 |
|  |  | *Oscarella* sp. 4 | MW016253 |
|  |  | *Oscarella* sp. 5 | MW016258 |
|  |  | *Oscarella* sp. 6 | MW016259 |
|  |  | *Oscarella* sp. 7 | MW016376 |
|  |  | *Plakina* sp. 1 | MW016264 |
|  |  | *Plakinastrella sp. 1* | MW016266 |
|  |  | *Plakortis* sp. 1 | MW016270 |

| Table S3  Additional sponge 28S rRNA barcodes deposited in GenBank. Taxonomic classification and GenBank accession numbers are provided. | | | | |
| --- | --- | --- | --- | --- |
|  |  |  |  |  |
| **Class** | **Order** | **Family** | **Species** | **GenBank Acc. No.** |
| Calcarea | Clathrinida |  | Leucettidae sp. 3 | MW016190 |
|  | Leucosolenida |  | Leucosolenida sp. 10 | PQ317792 |
|  |  |  | Leucosolenida sp. 22 | PQ317793 |
| Demospongiae | Axinellida |  | Axinellida sp. 1 | MW016051 |
|  | Biemnida |  | Bubarida sp. 1 | PQ317794 |
|  | Dendroceratida |  | Dendroceratida sp. 1 | MW016104 |
|  | Dictyoceratida |  | *Dysidea* sp. 3 | PQ317795 |
|  | Haplosclerida | Chalinidae | *Cladocroce* sp. 1 | PQ317796 |
|  |  |  | *Haliclona* sp. 23 | MW016142 |
|  |  |  | Haplosclerida sp. 8 | MW016168 |
|  |  |  | Haplosclerida sp. 12 | PQ317797 |
|  |  |  | Heteroscleromorpha sp. 3 | PQ317798 |
|  | Poecilosclerida | Coelosphaeridae | *Lissodendoryx* sp. 1 | MW016227 |
|  |  | Mycalidae | *Mycale* *parishii* | PQ317799 |
|  |  |  | *Mycale* sp. 4 | PQ317800 |
|  |  |  | *Mycale* sp. 14 | PQ317801 |
|  |  |  | Poecilosclerida sp. 2 | MW016275 |
|  |  |  | Poecilosclerida sp. 4 | PQ317802 |
|  |  |  | Poecilosclerida sp. 6 | MW016290 |
|  |  |  | Poecilosclerida sp. 10 | PQ317803 |
|  | Suberitida |  | Suberitida sp. 3 | PQ317805 |
|  |  |  | Suberitida sp. 4 | MW016364 |
|  |  | Suberitidae | Suberitidae sp. 4 | PQ317806 |
|  | Tethyida | Tethyidae | *Tethya* sp. 4 | PQ317807 |
| Homoscleromorpha | Homosclerophorida | Oscarellidae | *Oscarella* sp. 5 | PQ317808 |
|  | Homosclerophorida | Oscarellidae | *Oscarella* sp. 6 | PQ317809 |
|  | Homosclerophorida | Oscarellidae | *Oscarella* sp. 7 | PQ317810 |

| Table S4  Taxonomic identification swaps made in a custom R script, prioritizing the species known to be present in mock communities 1, 2, and 3 and in the ARMS unit. Those swaps were made when 1) species were tied for the top BLAST hit or when 2) the species identified in the top BLAST hit was a close relative to a species known to be present in the mix. | | | |
| --- | --- | --- | --- |
| **Order** | **Top BLAST hit identification** | **Swap (ties or close relatives)** | **Marker(s)** |
| Biemnida | *Biemna* sp. P60 | *Biemna fistulosa* | 28S |
| Bubarida | *Dictyonella* sp. SI_121Y | Bubarida sp. 1 JV-2020 | 28S |
| Clathrinida | Clathrinidae sp. 1 JV-2020 | Clathrinidae sp. 4 JV-2020 | 28S |
|  | Clathrinidae sp. 2 JV-2020 | Clathrinidae sp. 3 JV-2020 | 28S |
|  | Clathrinida sp. 2 JV-2020 | *Clathrinida* sp. 1 JV-2020 | 28S |
|  | Clathrinidae sp. 2 JV-2020 | Clathrinida sp. 2 JV-2020 | 28S |
|  | Clathrinidae sp. 3 JV-2020 | Clathrinidae sp. 4 JV-2020 | 28S |
|  | Clathrinidae sp. 8 JV-2020 | Clathrinidae sp. 4 JV-2020 | 28S |
|  | Clathrinidae sp. 11 JV-2020 | Clathrinidae sp. 4 JV-2020 | 28S |
| Clionaida | Clionaida sp. 1 JV-2020 | Clionaida sp. 2 JV-2020 | 28S |
| Leucosolenida | Leucosolenida sp. 10 JV-2020 | Leucosolenida sp. 7 JV-2020 | 28S |
| Haplosclerida | *Haliclona* sp. 23 JV-2020 | *Haliclona* sp. 1 JV-2020 | 28S |
|  | *Haliclona* laubenfelsi | *Haliclona* sp. 1 JV-2020 | 28S |
|  | Haplosclerida sp. 2 JV-2020 | *Haliclona* sp. 1 JV-2020 | 28S |
|  | Haplosclerida sp. 3 JV-2020 | *Haliclona* sp. 1 JV-2020 | 28S |
|  | Haplosclerida sp. 10 JV-2020 | *Haliclona* sp. 1 JV-2020 | 28S |
|  | Haplosclerida sp. 15 JV-2020 | *Haliclona* sp. 2 JV-2020 | 28S |
| Poecilosclerida | *Mycale* sp. SI_110Y | *Mycale* sp. 14 JV-2020 | 28S |
|  | Poecilosclerida sp. 5 JV-2020 | Poecilosclerida sp. 2 JV-2020 | COI |
| Suberitida | Suberitidae sp. 2 JV-2020 | Suberitidae sp. 1 JV-2020 | COI, 28S |
|  | Suberitidae sp. 3 JV-2020 | Suberitidae sp. 1 JV-2020 | COI |
|  | Suberitidae sp. 4 JV-2020 | Suberitidae sp. 1 JV-2020 | 28S |
|  | Suberitida sp. 4 JV-2020 | Suberitidae sp. 1 JV-2020 | 28S |
| Tethyida | *Tethya* sp. 1 JV-2020 | *Tethya* sp. 3 JV-2020 | 28S |
|  | *Tethya* sp. 2 JV-2020 | *Tethya* sp. 3 JV-2020 | 28S |
|  | *Tethya* sp. 4 JV-2020 | *Tethya* sp. 5 JV-2020 | 28S, COI |
|  | *Tethya* sp. 5 JV-2020 | *Tethya* sp. 3 JV-2020 | 28S |
|  | *Tethya* sp. 6 JV-2020 | *Tethya* sp. 5 JV-2020 | 28S |
|  | Tethyidae sp. 1 JV-2020 | *Tethya* sp. 5 JV-2020 | 28S |

| Table S5  Read abundance at different steps of the bioinformatics pipeline for all samples sequenced. | | | | | | | | | |
| --- | --- | --- | --- | --- | --- | --- | --- | --- | --- |
| **Cocktail** | **Sample** | **Read Abundance** | | | | | | | |
|  |  | **Raw demultiplexed data** | **Post-filtration** | **Denoised Forward** | **Denoised Reverse** | **Merged reads** | **No chimeras** | **Rarefied (for ARMS)** | **Post taxonomic assignment** |
| **Equimolar** | A1 | 63232 | 51043 | 50842 | 50905 | 48549 | 45749 | 45749 | 45514 |
|  | A2 | 63725 | 51883 | 51691 | 51719 | 49326 | 46317 | 46317 | 46083 |
|  | A3 | 67596 | 54902 | 54705 | 54744 | 52282 | 49146 | 49146 | 48897 |
|  | B | 276262 | 115591 | 114338 | 114619 | 63444 | 43985 | 43985 | 43311 |
|  | C | 271510 | 116198 | 114741 | 115143 | 61364 | 40710 | 40710 | 39727 |
| **% coverage** | A1 | 64180 | 52460 | 52378 | 52421 | 48856 | 45926 | 45926 | 45609 |
|  | A2 | 73911 | 60445 | 60331 | 60366 | 56373 | 52966 | 52966 | 52610 |
|  | A3 | 67596 | 54902 | 54705 | 54744 | 52282 | 49146 | 49146 | 48956 |
|  | B | 258806 | 107909 | 106840 | 107167 | 60489 | 45369 | 45369 | 44720 |
|  | C | 277260 | 116850 | 115951 | 116043 | 59172 | 45787 | 45369 | 45051 |
| **All OTUs** | - | 375400 | 163667 | 159015 | 160632 | 95485 | 75438 | 75438 | 74493 |
| **ARMS units** | 28S A1 | 716117 | 516760 | 514543 | 514789 | 465150 | 422131 | 98246 | 73659 |
|  | 28S A2 | 671273 | 489076 | 487477 | 487469 | 440613 | 402016 | 98246 | 75050 |
|  | 28S A3 | 717753 | 522545 | 520656 | 520755 | 470123 | 427503 | 98246 | 75189 |
|  | 28S B | 548253 | 208279 | 207176 | 206108 | 110176 | 98769 | 98769 | 80888 |
|  | 28S C | 521919 | 192878 | 189627 | 189523 | 112264 | 94159 | 71164 | 60427 |
|  | COI A | 140321 | 107787 | 104193 | 103042 | 98773 | 98246 | 98246 | 28115 |
|  | COI B | 194084 | 156919 | 149603 | 148555 | 140391 | 139866 | 98769 | 25158 |
|  | COI C | 158303 | 82590 | 79466 | 78668 | 74915 | 71164 | 71164 | 29671 |
| *Note:*  The raw reads for samples COI A, COI B, and COI C were obtained from Timmers et al., 2021. | | | | | | | | | |

| Table S6 Two-sample t-test comparison of unit-by-unit species richness between COI and 28S metabarcoding of complex coral reef communities on ARMS units. \| \| \| \|; T estimate \| df \| 95 % CI \| \| ρ; \| \| LL \| UL \|; -1.77 \| 3.65 \| -48.18 \| 11.52 \| 0.158; Sample mean estimate \| 28S : 41.67 COI : 60.00 \| \| \|; *Note.*  df = degrees of freedom; CI = confidence interval; LL = lower limit; UL = upper limit. \| \| \| \| Table S7 Read abundance of false positive sponge species detections in ARMS units communities for 28S and COI datasets. | | | | | | | | | | |
| --- | --- | --- | --- | --- | --- | --- | --- | --- | --- | --- |
| **Primer** | | **28S** | | | | **COI** | | | | |
| **Species** | | **ARMS unit A** | **ARMS unit B** | **ARMS unit C** | | **ARMS unit A** | **ARMS unit B** | | **ARMS unit C** | |
| *Calcinea* sp. 1 | | 3 | - | - | | - | - | | - | |
| *Callyspongia* sp. 2 | | - | - | 77 | | - | - | | - | |
| *Chelonaplysilla* erecta | | - | - | - | | - | 11 | | 6 | |
| *Cinachyra* sp. CJ-2016 | | - | - | - | | - | 9 | | - | |
| Clathrinidae sp. 8 | | - | - | 2 | | - | - | | - | |
| *Dendrilla* sp. FAR-2016 | | - | - | - | | - | - | | 297 | |
| *Dysidea* cf. *pallescens* | | - | - | 3 | | - | - | | - | |
| *Halichondria* *coerulea* | | - | - | - | | - | - | | 16 | |
| *Halichondria* sp. 1 | | - | - | - | | - | - | | 2 | |
| *Halichondria* sp. 2 | | - | - | 5 | | - | - | | - | |
| Haplosclerida sp. 8 | | - | - | 2 | | - | - | | - | |
| *Hymeniacidon* sp. 2 | | - | - | 2 | | - | 29 | | - | |
| *Ircinia* sp. 2 | | - | - | 3 | | - | - | | - | |
| *Lissodendoryx* sp. 1 | | - | - | 66 | | - | - | | - | |
| *Mycale cecilia* | | - | - | - | | 4 | - | | 3 | |
| *Mycale* sp. 10 | | - | - | 11 | | - | - | | - | |
| *Plakortis* sp. 1 | | - | - | - | | - | 74 | | - | |
| Poecilosclerida sp. 7 | | 9 | - | - | | - | - | | - | |
| *Protosuberites denhartogi* | | - | - | - | | - | 136 | | 2 | |
| *Racodiscula asteroides* | | 8 | 8 | 71 | | - | - | | - | |
| *Sigmaxinella hipposiderus* | | - | - | - | | 561 | 134 | | 16 | |
| *Spirastrella* sp. 1 | | - | - | 4 | | - | - | | - | |
| *Tedania* cf. *klausi* | | 6 | - | 25 | | - | - | | - | |
| *Tedania* sp. 1 | | - | - | 2 | | - | - | | - | |
| *Tethya* sp. 5 | | - | - | 3 | | - | - | | 4 | |
| Table S8 Generalized linear model of the relationship between image-based percent cover and read abundance of species in percent cover-based DNA concentration mock community 3. \| \| \| \| \|; Coefficients \| Estimate \| *SE* \| 95 % CI \| \| ρ; \| \| \| LL \| UL \|; Intercept \| 7.5837611 \| 0.2833324 \| 6.9769556153 \| 8.0957485 \| 2e-16 ***; Expected read values \| 0.0009946 \| 0.0002128 \| 0.0005302815 \| 0.0013836 \| 4.8e-05 ***; Dispersion parameter for quasipoisson \| 7208.824 \| \| \| \|; *Note.*  *SE* = standard error; CI = confidence interval; LL = lower limit; UL = upper limit. ***ρ = 0 \| \| \| \| \| Table S9 Generalized linear model of the relationship between image-based percent cover and read abundance of species in ARMS units communities. \| \| \| \| \|; Coefficients \| Estimate \| *SE* \| 95 % CI \| \| ρ; \| \| \| LL \| UL \|; Intercept \| 7.6105247 \| 0.4611319 \| 6.562381e+00 \| 8.405369763 \| 2e-16 ***; Expected read values \| 0.0009384 \| 0.4611319 \| 4.926918e-05 \| 0.001578271 \| 0.0136 *; Dispersion parameter for quasipoisson \| 19405.26 \| \| \| \|; *Note.*  *SE* = standard error; CI = confidence interval; LL = lower limit; UL = upper limit. ***ρ = 0 \| \| \| \| \| Table S10 Chi-square test of expected and observed read abundance for each sponge species for ARMS A | | | | | | | | | |  |
| **Species** | **Observed read abundance  (*O*)** | | | | **Expected read abundance (*E*)** | | | **Chi Squared (*O* – *E*)^2^/*E*** | |  |
| Ancorinidae sp. 3 | 2591 | | | | 3602 | | | 284 | |  |
| Clathrinida sp. 1 | 553 | | | |  |  |  | 2580 | |  |
| Clathrinidae sp. 4 | 11078 | | | |  |  |  | 15519 | |  |
| *Haliclona* sp. 1 | 20723 | | | |  |  |  | 81393 | |  |
| *Haliclona* sp. 2 | 4 | | | |  |  |  | 3594 | |  |
| Haplosclerida sp. 10 | 391 | | | |  |  |  | 2863 | |  |
| *Hymeniacidon* sp. 2 | 2724 | | | |  |  |  | 214 | |  |
| Leucosolenida sp. 1 | 1026 | | | |  |  |  | 1842 | |  |
| Leucosolenida sp. 3 | 941 | | | |  |  |  | 1965 | |  |
| Leucosolenida sp. 7 | 2135 | | | |  |  |  | 597 | |  |
| *Oscarella* sp. 6 | 22 | | | |  |  |  | 3558 | |  |
| Suberitidae sp. 1 | 2567 | | | |  |  |  | 297 | |  |
| *Tethya* sp. 5 | 2064 | | | |  |  |  | 657 | |  |
| Chi-Square Statistics (χ^2^) | 217184 | | | | | | | | |  |
| Degrees of freedom | 12 | | | | | | | | |  |
| P-value | < 0.01 | | | | | | | | |  |
| *Note.* The read abundance and Chi-Squared estimations have been rounded to the unit. | | | | | | | | | |  |

| Table S11  Chi-square test of expected and observed read abundance for each sponge species for ARMS B. | | | |
| --- | --- | --- | --- |
| **Species** | **Observed read abundance  (*O*)** | **Expected read abundance (*E*)** | **Chi Squared (*O* – *E*)^2^/*E*** |
| Ancorinidae sp. 3 | 410 | 2707 | 1949 |
| Clathrinida sp. 1 | 512 |  | 1779 |
| Clathrinidae sp. 3 | 1094 |  | 961 |
| Clathrinidae sp. 4 | 2685 |  | 0 |
| Dendroceratida sp. 3 | 215 |  | 2294 |
| *Halichondria* sp. 1 | 6157 |  | 4399 |
| *Haliclona* sp. 1 | 10409 |  | 21922 |
| *Haliclona* sp. 2 | 16 |  | 2675 |
| *Ircinia* sp. 2 | 1028 |  | 1041 |
| Leucosolenida sp. 1 | 436 |  | 1905 |
| Leucosolenida sp. 5 | 599 |  | 1641 |
| Leucosolenida sp. 7 | 863 |  | 1256 |
| Poecilosclerida sp. 2 | 2757 |  | 1 |
| Suberitidae sp. 1 | 9010 |  | 14682 |
| *Tethya* sp. 3 | 2336 |  | 51 |
| *Tethya* sp. 5 | 882 |  | 1230 |
| **Chi-Square Statistics (χ^2^)** | 246901 | | |
| **Degrees of freedom** | 15 | | |
| **P-value** | < 0.01 | | |
| *Note.* The read abundance and Chi-Squared estimations have been rounded to the unit. | | | |

| Table S12  Chi-square test of expected and observed read abundance for each sponge species for ARMS C. | | | |
| --- | --- | --- | --- |
| Species | Observed read abundance  (*O*) | Expected read abundance (*E*) | Chi Squared (*O* – *E*)^2^/*E* |
| Ancorinidae sp. 3 | 334 | 2207 | 1589 |
| *Aplysilla rosea* | 31 |  | 2145 |
| Clathrinidae sp. 3 | 885 |  | 791 |
| Clathrinidae sp. 4 | 2518 |  | 44 |
| Clathrinidae sp. 5 | 828 |  | 861 |
| *Dysidea* sp. 5 | 3665 |  | 964 |
| *Haliclona* sp. 1 | 9835 |  | 26374 |
| Leucosolenida sp. 1 | 559 |  | 1230 |
| Leucosolenida sp. 11 | 2431 |  | 23 |
| Leucosolenida sp. 13 | 0 |  | 2207 |
| Leucosolenida sp. 5 | 761 |  | 947 |
| Leucosolenida sp. 7 | 1423 |  | 278 |
| *Plakina* sp. 1 | 76 |  | 2057 |
| Poecilosclerida sp. 1 | 2272 |  | 2 |
| Poecilosclerida sp. 2 | 505 |  | 1312 |
| Suberitidae sp. 1 | 2217 |  | 0 |
| Suberitidae sp. 3 | 10634 |  | 32188 |
| *Tethya* sp. 3 | 701 |  | 1027 |
| Chi-Square Statistics (χ^2^) | 244952 | | |
| Degrees of freedom | 17 | | |
| P-value | < 0.01 | | |
| *Note.* The read abundance and Chi-Squared estimations have been rounded to the unit. | | | |

| Table S13  Fold deviation from the expected number of reads for the Equimolar cocktail. | | | | | |
| --- | --- | --- | --- | --- | --- |
| **Taxa** | | | **fold deviation from expected number of reads (observed – expected/ expected)** | | |
| **Sponge class** | **Sponge order** | **Sponge species** | **Unit A** | **Unit B** | **Unit C** |
| Calcarea | Clathrinida | Clathrinida sp. 1 | -0.85 | -0.81 | - |
|  |  | Clathrinidae sp. 5 | - | - | -0.62 |
|  |  | Clathrinidae sp. 3 | - | -0.6 | -0.6 |
|  |  | Clathrinidae sp. 4 | 2.08 | -0.01 | 0.14 |
|  | Leucosolenida | Leucosolenida sp. 1 | -0.72 | -0.84 | -0.75 |
|  |  | Leucosolenida sp. 3 | -0.74 | - | - |
|  |  | Leucosolenida sp. 5 | - | -0.78 | -0.66 |
|  |  | Leucosolendia sp. 7 | -0.41 | -0.68 | -0.36 |
|  |  | Leucosolendia sp. 11 | - | - | 0.1 |
|  |  | Leucosolendia sp. 13 | - | - | -1 |
| Demospongiae | Dendroceratida | *Aplysilla rosea* | - | - | -0.99 |
|  |  | Dendroceratida sp. 3 | - | -0.92 | - |
|  | Dictyoceratida | *Dysidea* sp. 5 | - | - | 0.66 |
|  |  | *Ircinia* sp. 2 | - | -0.62 | - |
|  | Haplosclerida | *Haliclona* sp. 1 | 4.75 | 2.85 | 3.46 |
|  |  | *Haliclona* sp. 2 | -0.99 | -0.99 | - |
|  |  | Haplosclerida sp. 10 | -0.89 | - | - |
|  | Poecilosclerida | Poecilosclerida sp. 1 | - | - | 0.03 |
|  |  | Poecilosclerida sp. 2 | - | 0.02 | - |
|  |  | Poecilosclerida sp. 7 | - | - | -0.77 |
|  | Suberitida | *Halichondria* sp. 1 | - | 1.27 | - |
|  |  | *Hymeniacidon* sp. 2 | -0.24 | - | - |
|  |  | Suberitidae sp. 1 | -0.29 | 2.33 | 3.82 |
|  |  | Suberitida sp. 3 | - | - | 0 |
|  | Tethyida | *Tethya* sp. 3 | - | -0.14 | -0.68 |
|  |  | *Tethya* sp. 5 | -0.43 | -0.67 | - |
|  | Tetractinellida | Ancorinidae sp. 3 | -0.28 | -0.85 | -0.85 |
| Homoscleromorpha | Homosclerophorida | *Oscarella* sp. 6 | -0.99 | - | - |
|  |  | *Plakina* sp. 1 | - | - | -0.97 |
| *Note.*  Fold deviations were rounded to two decimals. | | | | | |
